# Transition‐Metal Chemistry of Alkaline‐Earth Elements: The Trisbenzene Complexes M(Bz)_3_ (M=Sr, Ba)

**DOI:** 10.1002/anie.201908572

**Published:** 2019-10-17

**Authors:** Qian Wang, Sudip Pan, Yan‐Bo Wu, Guohai Deng, Jian‐Hong Bian, Guanjun Wang, Lili Zhao, Mingfei Zhou, Gernot Frenking

**Affiliations:** ^1^ Department of Chemistry, Collaborative Innovation Center of Chemistry for Energy Materials Shanghai Key Laboratory of Molecular Catalysis and Innovative Materials Fudan University Shanghai 200433 China; ^2^ Institute of Advanced Synthesis School of Chemistry and Molecular Engineering Jiangsu National Synergetic Innovation Center for Advanced Materials Nanjing Tech University Nanjing 211816 China; ^3^ Institute of Molecular Science Shanxi University Taiyuan 030006 China; ^4^ Fachbereich Chemie Philipps-Universität Marburg Hans-Meerwein-Strasse 4 35043 Marburg Germany

**Keywords:** 18-electron rule, alkaline earth metals, benzene complexes, bonding analysis, matrix isolation spectroscopy

## Abstract

We report the synthesis and spectroscopic identification of the trisbenzene complexes of strontium and barium M(Bz)_3_ (M=Sr, Ba) in low‐temperature Ne matrix. Both complexes are characterized by a *D*_3_ symmetric structure involving three equivalent η^6^‐bound benzene ligands and a closed‐shell singlet electronic ground state. The analysis of the electronic structure shows that the complexes exhibit metal–ligand bonds that are typical for transition metal compounds. The chemical bonds can be explained in terms of weak donation from the π MOs of benzene ligands into the vacant (n−1)d AOs of M and strong backdonation from the occupied (n−1)d AO of M into vacant π* MOs of benzene ligands. The metals in these 20‐electron complexes have 18 effective valence electrons, and, thus, fulfill the 18‐electron rule if only the metal–ligand bonding electrons are counted. The results suggest that the heavier alkaline earth atoms exhibit the full bonding scenario of transition metals.

## Graphical abstract

**Bonding scenarios**: The synthesis and spectroscopic identification of the trisbenzene complexes of strontium and barium M(Bz)_3_ (M=Sr, Ba) in a low‐temperature Ne matrix is reported. The complexes exhibit metal–ligand bonds that are typical for transition‐metal compounds. The results suggest that the heavier alkaline‐earth atoms exhibit the full bonding scenario of transition metals.

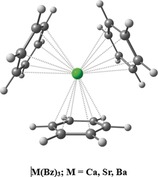

## Introduction

The alkali metals and the alkaline earth elements with a (n)s^1^ or (n)s^2^ valence shell configuration, respectively, are the most electropositive elements in the periodic table. These classical s‐block main group elements were for a long time considered to be among the rather uninteresting elements in terms of chemical bonding due to preconceived notions about their valence orbitals, their reactivity and handling.^1^ They readily lose the outermost (n)s electron(s) to form ionic compounds in the +1 and +2 oxidation states. But recent reports showed that the alkali and alkaline earth elements have a much richer chemistry than hitherto thought.^2^, ^3^, ^4^, ^5^, ^6^, ^7^, ^8^, ^9^, ^10^, ^11^ In particular, the heavier alkaline earth atoms M=Ca, Sr, Ba may use their (n−1)d functions as genuine valence orbitals in the octa‐coordinated complexes M(CO)_8_ 7 and M(N_2_)_8_.^11^ The cubic (*O*_h_) adducts possess metal–ligand bonds that are typical for transition‐metal complexes. The participation of the (n−1)d atomic orbitals (AOs) of barium in chemical bonding had been suggested already in earlier theoretical studies^12^, ^13^, ^14^, ^15^, ^16^, ^17^ and barium was even called by Pyykkö an “honorary transition metal”.^17^ Recent investigations demonstrated that (n−1)d AO bonding is not restricted to Ba only and that the heavier atoms Ca, Sr, Ba exhibit a transition metal chemistry to a much larger extent than ever known so far.^7^, ^8^, ^9^, ^10^, ^11^ Here, we report the isolation and spectroscopic identification of the trisbenzene complexes of heavier alkaline earth atoms M(Bz)_3_ (M=Sr, Ba). The synthesis of the first transition metal benzene complex Cr(Bz)_2_ by Fischer and Hafner in 1955 was a milestone in transition metal chemistry.^18^ The isolation of Sr(Bz)_3_ and Ba(Bz)_3_ now opens the door to another chapter of metal complexes.

## Results and Discussion

**Figure 1:**
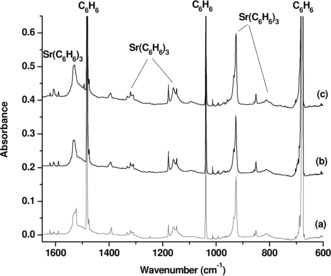
Infrared absorption spectra of strontium–benzene complexes in the 1650–600 cm^−1^ region from co‐deposition of laser‐evaporated strontium atoms with 0.1 % C_6_H_6_ in neon. a) 30 min of sample deposition at 4 K, b) after annealing at 12 K, c) after 20 min of visible light irradiation.

The strontium and barium–benzene complexes were prepared by co‐deposition of laser‐evaporated strontium and barium atoms with benzene molecules in excess neon at 4 K.^19^ The experiments were performed using relatively low laser energy to avoid the formation of multinuclear species. A series of experiments were performed using different benzene concentrations ranging from 0.025–0.5 %. The IR spectra in the 1650–600 cm^−1^ region from co‐deposition of laser‐evaporated strontium atoms with 0.1 % C_6_H_6_ are represented in Figure 1.

**Figure 2:**
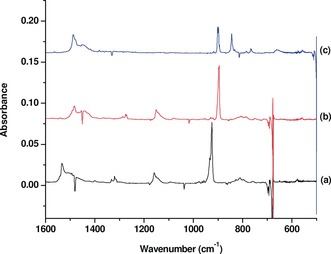
Difference spectra of strontium–benzene complexes in the 1600–500 cm^−1^ region from co‐deposition of laser‐evaporated strontium atoms with isotopic‐labeled benzene in excess neon (the spectrum taken after 20 min of visible light irradiation minus the spectrum taken after 12 K annealing). a) 0.1 % C_6_H_6_, b) 0.2 % ^13^C_6_H_6_, and c) 0.2 % C_6_D_6_.

A group of metal‐dependent product absorptions are observed after sample deposition at 4 K, remain almost unchanged on sample annealing to 12 K and increase under 20 min of visible light excitation using a high pressure mercury arc lamp with a 495 nm long‐wavelength pass filter (*λ*=495–600 nm). Since some bands are partially overlapped by the benzene absorptions, a difference spectrum taken from the spectrum after visible light excitation minus the spectrum after 12 K annealing is obtained, as shown in Figure 2. The upward bands represent the product absorptions, while the downward bands are due to benzene absorptions. It clearly shows the formation of the product absorptions with the consumption of the benzene absorptions. The bands are quite broad and some bands show site absorptions. Laser evaporation of alkaline earth metal targets might easily lead to formation of cations. In order to verify whether the observed species is a cation or a neutral, additional experiments were performed by adding a trace of CCl_4_ in the reactant gas to serve as an electron trap.^20^ CCl_4_ captures most of electrons produced by laser ablation during sample deposition, and thus would facilitate the survival of more cation products. The spectra from the experiment with 0.01 % CCl_4_ added to the neon matrix gas are compared to the spectra from the experiment without CCl_4_ doping in Figure S1 of Supporting Information. The results show that the observed product absorptions are reduced relative to other absorptions, indicating that these product absorptions are due to a neutral species rather than a cation.

**Figure 3:**
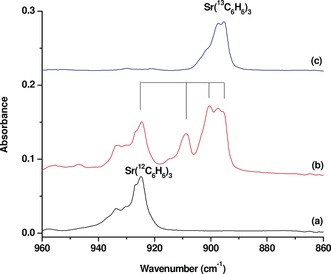
Infrared absorption spectra of strontium–benzene complexes in the 960‐860 cm^−1^ region from co‐deposition of laser‐evaporated strontium atoms with isotopic‐labeled benzene in excess neon (the spectra were taken 20 min of visible light irradiation). a) 0.1 % C_6_H_6_, b) 0.15 % ^12^C_6_H_6_ + 0.15 % ^13^C_6_H_6_, and c) 0.2 % ^13^C_6_H_6_.

Isotopic substitution experiments (^13^C_6_H_6_, C_6_D_6_, and ^12^C_6_H_6_ + ^13^C_6_H_6_) were performed for product identification based on isotopic shifts and absorption splitting. As shown in Figure 2, all the absorptions are shifted with the ^13^C_6_H_6_ and C_6_D_6_ samples. The assignment of these product absorptions to the tris(benzene)strontium complex is based on the isotopic splitting of the most intense band at 924.9 cm^−1^, which is due to the benzene ring breathing mode based on the isotopic data. The spectrum from the experiment using an equimolar mixture of ^12^C_6_H_6_ and ^13^C_6_H_6_ shows that a quartet with two weak intermediates is produced (The low frequency intermediate band is partially overlapped by the strong absorption of Sr(^13^C_6_H_6_)_3_, See Figure 3). This mixed isotopic spectral feature indicates that the 924.9 cm^−1^ band is due to a doubly degenerate benzene ring breathing mode of a tris(benzene) complex involving three equivalent benzene ligands. The positions and mode assignment of the observed bands are listed in Table 1. Besides the most intense ring breathing mode, the second most intense band at 1534.8 cm^−1^ is assigned to the C−C stretching mode. The third strongest band at 1159.1 cm^−1^ is attributed to the C−H in‐plane bending mode. The remaining bands are weak. The bands at 1332.6 and 1320.1 cm^−1^ are assigned to mixed modes involving C−C stretching and in‐plane C−H bending. The bands at 933.8 and 810.7 cm^−1^ are attributed to the out‐of‐plane C−H bending modes.

**Table 1:** Experimental infrared absorptions (cm^−1^) of Sr(C_6_H_6_)_3_, Sr(^13^C_6_H_6_)_3_ and Sr(C_6_D_6_)_3_ in solid neon and calculated values at the M06‐2X(D3)/def2‐TZVPP level (*D*_3_ symmetry). Calculated IR intensities (km mol^−1^) are given in parentheses.

Mode	Experimental					Calculated				
	^12^C_6_H_6_	^13^C_6_H_6_	Δ^[a]^	^12^C_6_D_6_	Δ^[a]^	^12^C_6_H_6_	^13^C_6_H_6_	Δ^[a]^	^12^C_6_D_6_	Δ^[a]^
C=C stretch	1534.8	1483.0	−51.8	1487.6	−47.2	1556.8 (700)	1506.8 (608)	−50.0	1504.1 (812)	−52.7
C=C stretch and C‐D in‐plane bend				1382.1					1399.6 (54)	
C=C stretch and C‐D in‐plane bend				1366.0					1372.2 (21)	
C=C stretch and C−H in‐plane bend	1332.6	1286.4	−46.2			1365.3 (49)	1317.5 (47)	−47.8		
C=C stretch and C−H in‐plane bend	1320.1	1274.2	−45.9			1346.4 (34)	1299.7 (32)	−46.7		
C−H(D) in‐plane bend	1159.1	1151.6	−7.5	844.6	−314.5	1173.3 (119)	1163.4 (155)	−9.9	860.8 (50)	−312.5
C−H(D) in‐plane bend	1094.6	1080.6	−14.0	832.6	−262.0	1141.6 (15)	1129.0 (17)	−12.6	848.5 (65)	−293.1
C−H(D) out‐of‐plane bend	933.8			766.8	−167.0	993.6 (5)			809.0 (53)	−184.6
Ring breath	924.9	895.5	−29.4	898.1	−26.8	968.0 (415)	935.4 (374)	−32.6	936.8 (203)	−31.2
C−H(D) out‐of‐plane bend	810.7	803.1	−7.6	662.0	−148.7	853.1 (58)	845.5 (53)	−7.6	690.2 (50)	−162.9

[a] Shift with respect to ^12^C_6_H_6_.

The Sr(C_6_H_6_)_3_ absorptions are the only product absorptions observed in the experiments using different benzene concentrations as well as laser energy. The spectra from experiments using different laser energy are shown in Figure S2. There is no any experimental evidence of formation of the Sr(C_6_H_6_) and Sr(C_6_H_6_)_2_ complexes in these experiments.

**Figure 4:**
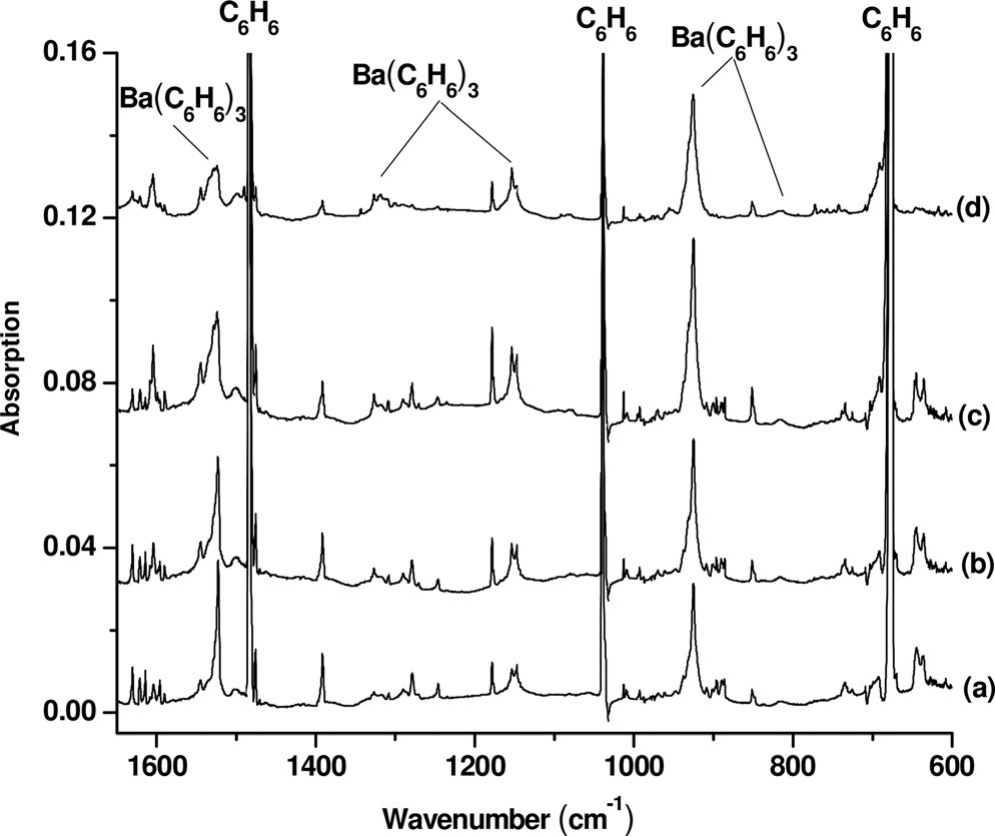
Infrared absorption spectra of barium–benzene complexes in the 1650–600 cm^−1^ region from co‐deposition of laser‐evaporated barium atoms with 0.1 % C_6_H_6_ in neon. a) 30 min of sample deposition at 4 K, b) after annealing at 10 K, c) after annealing to 12 K, and d) after 20 min of UV‐visible light irradiation.

Similar absorptions are also observed in the barium experiments. The IR spectra are shown in Figure 4. All the absorptions are observed on sample deposition and increase on sample annealing to 10 K and 12 K, and remain almost unchanged under visible light irradiation (*λ*=495–600 nm), but decrease upon UV‐visible light irradiation using the high pressure mercury arc lamp without filters (*λ*=250–600 nm). The band positions are only slightly blue‐ or red‐shifted with respect to the corresponding modes of tris(benzene)strontium (within 10 cm^−1^ difference). The spectra from the isotopic‐labeled experiments are shown in Figures S3 and S4. The band positions are listed in Table 2. No similar product absorptions are observed in the experiments with calcium.

**Table 2:** Experimental infrared absorptions (cm^−1^) of Ba(C_6_H_6_)_3_, Ba(^13^C_6_H_6_)_3_ and Ba(C_6_D_6_)_3_ in solid neon and calculated values at the M06‐2X(D3)/def2‐TZVPP level (*D*_3_ symmetry). Calculated IR intensities (km mol^−1^) are given in parentheses.

Mode	Experimental					Calculated				
	^12^C_6_H_6_	^13^C_6_H_6_	Δ^[a]^	^12^C_6_D_6_	Δ^[a]^	^12^C_6_H_6_	^13^C_6_H_6_	Δ^[a]^	^12^C_6_D_6_	Δ^[a]^
C=C stretch	1529.0	1479.5	−49.5	1486.7	−42.3	1575.0 (651)	1523.7 (562)	−51.3	1523.1 (769)	−51.9
C=C stretch and C‐D in‐plane bend				1382.2					1438.9 (124)	
C=C stretch and C‐D in‐plane bend				1360.7					1386.8 (50.2)	
C=C stretch and C−H in‐plane bend	1327.1	1281.9	−45.2			1360.3 (76)	1312.0 (70)	−48.3		
C=C stretch and C−H in‐plane bend	1318.4	1272.2	−46.2			1343.6 (18)	1296.9 (17)	−46.7		
C−H(D) in‐plane bend	1153.6	1145.0	−8.6	845.2	−308.4	1172.7 (152)	1163.1 (180)	−9.6	858.4 (40)	−314.3
C−H(D) in‐plane bend	1084.6	1073.0	−11.6	838.7	−245.9	1144.1 (2)	1130.5 (3)	−13.6	853.8 (98)	−290.3
C−H(D) out‐of‐plane bend	932.2			766.9	−165.3	986.5 (8)			801.5 (70)	−185.0
Ring breath	925.8	894.8	−31	899.2	−26.6	969.4 (472)	937.0 (430)	−32.4	937.8 (224)	−31.6
C−H(D) out‐of‐plane bend	813.8	806.8	−7.0	665.1	−148.7	831.2 (11)	824.3 (11)	−6.9	659.4 (4)	−171.8

[a] Shift with respect to ^12^C_6_H_6_.

We calculated the trisbenzene complexes M(Bz)_3_ (M=Ca, Sr, Ba) and analyzed their electronic structures with quantum chemical methods using density functional theory (DFT) at the M06‐2X(D3)/def2‐TZVPP level of theory, which considers also dispersion interactions. Details of the methods are given in Supporting Information. The calcium complex was also included in the theoretical work in order to provide a comparison with the experimentally observed strontium and barium adducts.

**Figure 5:**
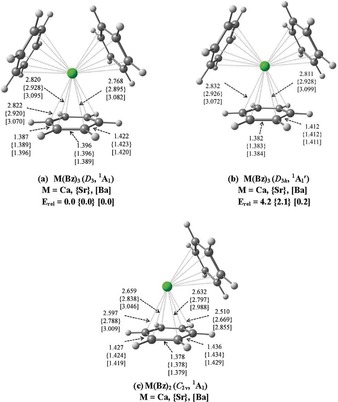
Calculated geometries of M(Bz)_3_ and M(Bz)_2_ complexes (M=Ca, Sr, Ba) at M06‐2X‐D3/def2‐TZVPP. Bond distances are in Å, Relative energies are in kcal mol^−1^.

The geometry optimizations give *D*_3_ symmetric M(Bz)_3_ equilibrium structures with a singlet (^1^A_1_) electronic state as energy minima (Figure 5 a). The benzene ligands are distorted from their *D*_6*h*_ geometry in the free molecules to *C*_2_ symmetric fragments with two C−C distances being significantly longer (1.420–1.423 Å) than in free benzene (1.388 Å), while the other C−C bond lengths are only slightly altered. Calculations with enforced *D*_3*h*_ symmetry give structures being marginally higher in energy than the *D*_3_ form (Figure 5 b). The *D*_3*h*_ structures of Ca(Bz)_3_ and Sr(Bz)_3_ have 3 imaginary frequencies, but the *D*_3*h*_ form of Ba(Bz)_3_ is calculated as energy minimum, which is only 0.2 kcal mol^−1^ less stable than the *D*_3_ structure. The *D*_3*h*_ structures of M(Bz)_3_ have four C−C bonds that are clearly longer (1.411–1.412 Å) than in free benzene while two C−C bonds are a bit shortened (1.382–1.384 Å). The corresponding triplet states of M(Bz)_3_ posses *C*_1_ symmetry for M=Ca and *C*_2_ symmetry for M=Sr, Ba and they are 0.6–3.8 kcal mol^−1^ higher in energy than the singlet *D*_3_ structures (see Figure S5).

Table 3 shows the bond dissociation energies (BDEs) of M(Bz)_3_ for the loss of one or all three benzene ligands. The calculations suggest that the BDE of one ligand ranges between *D*_e_=19.4 kcal mol^−1^ for M=Ca and *D*_e_=26.1 kcal mol^−1^ for M=Sr. The BDEs at room temperature become smaller after correcting for thermodynamic contributions, having values between Δ*G* ^298^=6.5 kcal mol^−1^ (Ca) and Δ*G* ^298^=14.2 kcal mol^−1^ (Sr). The total BDEs for fragmentation into three benzene molecules are between *D*_e_=38.6 kcal mol^−1^ (Ca) and *D*_e_=46.0 kcal mol^−1^ (Ba), but the free energies at room temperature are significantly smaller having values between Δ*G* ^298^=8.7 kcal mol^−1^ (Ca) and Δ*G* ^298^=19.5 kcal mol^−1^ (Ba). Note that the latter process includes the electronic relaxation of the metal atoms to the ^1^S electronic ground state. The lower BDE of the calcium complex may be the reason why Ca(Bz)_3_ could not be observed experimentally.

**Table 3:** Calculated bond dissociation energies of M(Bz)_3_ complexes for the loss of one Bz and three Bz molecules at the M06‐2X‐D3/def2‐TZVPP level. The *D*_e_ values give the electronic energies, the *D*_0_ data include the corrections by zero‐point vibrational frequencies and the Δ*G* ^298K^ values consider the thermodynamic and vibrational corrections at room temperature.

Reaction	M	*D*_e_	*D*_0_	Δ*G* ^298K^
M(Bz)_3_ (*D*_3_, ^1^A_1_) → M(Bz)_2_ (*C*_2*v*_, ^1^A_1_) + Bz	Ca	19.4	18.3	6.5
	Sr	26.1	25.3	14.2
	Ba	24.0	23.6	13.4
M(Bz)_3_ (*D*_3_, ^1^A_1_) → M (^1^S) + 3 Bz	Ca	38.6	38.3	8.7
	Sr	39.2	39.7	11.0
	Ba	46.0	47.1	19.5

The calculated infrared signals of the M(Bz)_3_ complexes and their ^13^C and deuterated isotopes in the region >600 cm^−1^ as well as the frequency shifts are shown in Tables 1 and 2. The agreement of the computed unscaled values of the harmonic frequencies with the experimental values of the anharmonic mode is quite good. The calculated isotopic frequency shifts are also in very good accord with the recorded spectra. The comparison between the calculated and observed IR spectra leaves no doubt that the experimentally observed species are the neutral trisbenzene complexes M(Bz)_3_ (M=Sr, Ba). The radical cations [M(Bz)_3_]^+^ (M=Sr, Ba) were also calculated. The optimized structures are shown in Figure S6. Both cations are predicted to have D_3_ symmetric structures with a doublet (^2^A_1_) electronic ground state. The calculated vibrational frequencies and intensities are listed in Tables S1 and S2, respectively. Both cations are predicted to have two intense well‐separated C=C stretching modes. Therefore, the assignment of the experimentally observed species to the radical cations can clearly be ruled out, as only one C=C stretching band is observed experimentally.

The complexes M(Bz)_3_ (M=Ca, Sr, Ba) are formally 20‐electron species possessing a singlet (^1^A_1_) electronic ground state. Our previously reported isoelectronic alkaline earth complexes M(CO)_8_ 7 and M(N_2_)_8_ 11 are 18‐electron adducts with cubic (*O*_h_) symmetry that possess a triplet (^3^A_1g_) ground state. The latter molecules have two singly occupied degenerate orbitals and one lower lying ligand‐based molecular orbital (MO) with a_2u_ symmetry that has zero coefficient at the metal, because it has no valence AO having proper symmetry. We earlier reported the observation of the 20‐electron transition metal octa‐carbonyl anions [TM(CO)_8_]^−^ (TM=Sc, Y, La).^21^ These complexes also have cubic symmetry but a singlet (^1^A_1g_) ground state, where the degenerate HOMO is fully occupied. The 18‐electron rule first suggested by Langmuir^22^ remains valid in the above complexes, if only the metal–ligand bonding electrons are counted.

**Figure 6:**
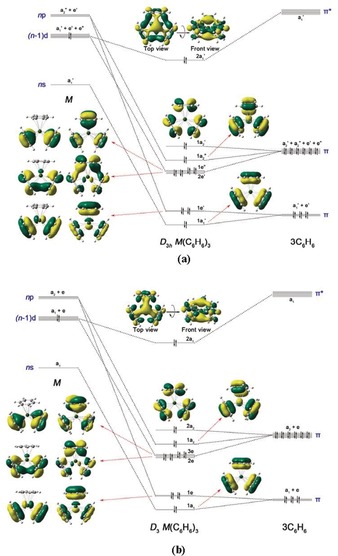
MO correlation diagram of the valence (n)s(n)p(n−1)d AOs of M and (C_6_H_6_)_3_ in a) *D*_3*h*_ symmetry and b) *D*_3_ symmetry. Shapes of the MOs of Ba(Bz)_3_ are also shown.

Figure 6 a shows the orbital correlation diagram for the splitting of the (n)s(n)p(n−1)d AOs of alkaline earth atoms in the *D*_3*h*_ field of three benzene ligands along with plots of the associated occupied MOs of M(Bz)_3_. In the *D*_3*h*_ symmetry field, the five d orbitals of metal split into three irreducible representations (a_1_′ + e′ + e′′). The a_1_′ orbital, which is the d${}_{z{}^{2}}$ AO of the metal, provides backdonation to the antibonding π* orbital of the benzene ligands leading to the bonding MO (2a_1_′). The remaining eight metal AOs serve as acceptor orbitals for the charge donation from the bonding π orbitals of the benzene ligands. There are nine symmetry‐adapted linear combination orbitals from the bonding π orbitals of three benzene ligands. The 1a_2_′ MO remains as a ligand‐only orbital, because there is no valence AO of the metal possessing this symmetry. All of these bonding and nonbonding orbitals are doubly occupied, resulting in a closed‐shell singlet ground state. Thus, the M(Bz)_3_ complexes have a total of 20 valence electrons, but the metal centers have only 18 effective valence electrons, and thus still fulfill the 18‐electron rule if only the metal–ligand bonding electrons are considered.

Figure 6 b shows the related orbital diagram for M(Bz)_3_ in the *D*_3_ field of the benzene ligands at the equilibrium geometries and the shape of the occupied MOs of M(Bz)_3_. Comparing the latter MOs with the *D*_3*h*_ orbitals shows only negligible differences. The 2a_2_ MO of the *D*_3_ form takes the position of the 2a_2_′ MO of the *D*_3*h*_ form and could, in principle, mix with one (n)p AO of the metal. Inspection of the MO coefficients shows that the contribution of the (n)p AO of the metal is nearly zero. Thus, the pattern of the orbital interaction of the *D*_3_ structures is very similar to the *D*_3*h*_ form. We think that the deviation from perfect *D*_3*h*_ geometry of the M(Bz)_3_ complexes is due to weak steric interactions between the benzene ligands, which have a negligible effect on the orbital interactions. This corresponds to the reduction of the energy difference between the *D*_3_ and *D*_3*h*_ forms of M(Bz)_3_ having the order of Ca>Sr>Ba, because the metal–ligand bonds and thus the ligand–ligand distances increase (Figure 5).

On the basis of above bonding analysis, it is clear that the M(Bz)_3_ complexes can be regarded as being formed via the interactions between the metal atoms in the singlet excited state with an (n−1)d^2^ns^0^np^0^ electron configuration and three benzene ligands in the singlet state. The strength of the pairwise orbital interactions can be estimated with the EDA‐NOCV (energy decomposition analysis in combination with natural orbitals for chemical valence) method.^23^ Details of the method have been described in the literature.^24^, ^25^, ^26^ The calculations at M06‐2X with the Slater basis functions TZ2P of the ADF program for M in the singlet excited state with d^2^ configuration did not converge. Therefore, we used the BP86‐D3(BJ) functional in conjunction with the TZ2P basis set. Our experience in numerous studies has shown that the EDA‐NOCV calculations are not very sensitive to the functional.^27^, ^28^, ^29^, ^30^, ^31^, ^32^, ^33^, ^34^, ^35^, ^36^, ^37^ Table 4 gives the numerical results of the calculations of M(Bz)_3_ (M=Ca, Sr, Ba) at the *D*_3_ equilibrium structures.

**Table 4:** Results of EDA‐NOCV calculations at BP86‐D3(BJ)/TZ2P for M(Bz)_3_ (*D*_3_, ^1^A_1_) complexes using neutral atoms M in the singlet state with ns^0^np^0^(n−1)d^2^ electron configuration and (Bz)_3_ (singlet) as interacting fragments.

Term	Interaction	Ca (S, 4s^0^4p^0^3d^2^) + (Bz)_3_ (S)	Sr (S, 5s^0^5p^0^4d^2^) + (Bz)_3_ (S)	Ba (S, 6s^0^6p^0^5d^2^) + (Bz)_3_ (S)
Δ*E*_int_		−197.4	−191.5	−135.5
Δ*E*_Pauli_		124.5	140.5	156.8
Δ*E*_disp_^[a]^		−7.1 (2.2 %)	−8.4 (2.5 %)	−16.8 (5.7 %)
Δ*E*_elstat_^[a]^		−82.7 (25.7 %)	−88.8 (26.8 %)	−116.4 (39.8 %)
Δ*E*_orb_^[a]^		−232.1 (72.1 %)	−234.7 (70.7 %)	−159.1 (54.4 %)
Δ*E*_orb(1)_^[b]^ (2a_1_)	(Bz)_3_←M(d) backdonation	−191.8 (82.6 %)	−189.9 (80.9 %)	−119.9 (75.4 %)
Δ*E*_orb(2)_^[b]^ (3e)	(Bz)_3_→M(d) donation	−14.0 (6.0 %)	−16.8 (7.2 %)	−16.8 (10.6 %)
Δ*E*_orb(3)_^[b]^ (2e)	(Bz)_3_→M(d) donation	−13.6 (5.9 %)	−15.0 (6.4 %)	−11.8 (7.4 %)
Δ*E*_orb(4)_^[b]^ (1a_1_)	(Bz)_3_→M(s) donation	−2.7 (1.2 %)	−2.9 (1.2 %)	−2.1 (1.3 %)
Δ*E*_orb(5)_^[b]^ (1a_2_)	(Bz)_3_→M(p) donation	−1.4 (0.6 %)	−1.3 (0.6 %)	−0.8 (0.5 %)
Δ*E*_orb(6)_^[b]^ (1e)	(Bz)_3_→M(p) donation	−3.2 (1.4 %)	−2.4 (1.0 %)	−0.8 (0.5 %)
Δ*E*_orb(7)_^[b]^ (1a_2_)	Polarization	−1.3 (0.6 %)	−1.8 (0.8 %)	−2.5 (1.6 %)
Δ*E*_orb(rest)_^[b]^		−4.1 (1.8 %)	−4.6 (2.0 %)	−4.4 (2.8 %)

[a] The values in parentheses give the percentage contribution to the total attractive interactions Δ*E*_elstat_ + Δ*E*_orb_ + Δ*E*_disp_. [b] The values in parentheses give the percentage contribution to the total orbital interactions Δ*E*_orb_.

The data in Table 4 suggest that the M‐(Bz)_3_ attractive interactions come mainly from the covalent term Δ*E*_orb_, which provides between 72 % (M=Ca) and 54 % (M=Ba) to the total attraction. The breakdown of Δ*E*_orb_ into pairwise orbital interactions shows that the dominant contribution Δ*E*_orb(1)_ comes from the backdonation of the occupied (n−1)d AO of the metal into the vacant π* orbitals of the ligands denoted as (Bz)_3_←M(d). This orbital interaction contributes between 83 % (M=Ca) and 75 % (M=Ba) to Δ*E*_orb_. The following terms Δ*E*_orb(2)_ and Δ*E*_orb(3)_ also involve the (n−1)d AOs of the metals. They are due to the donation from two sets of degenerate occupied π MOs of the ligands into vacant (n−1)d metal AOs denoted as (Bz)_3_→M(d). Thus, the valence AOs of the metals that contribute to the metal–ligand bonding are dominated by the (n−1)d orbitals.

**Figure 7:**
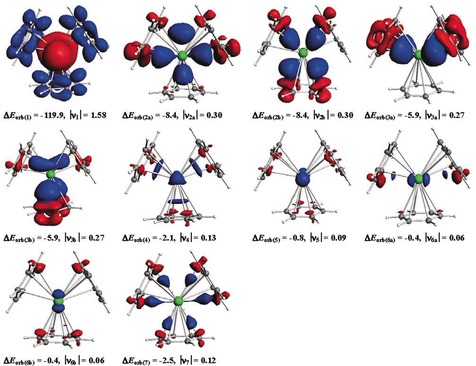
Shape of the deformation densities Δ*ρ*_(1)‐(7)_, which are associated with the orbital interactions Δ*E*_orb(1)‐(7)_ between neutral fragments Ba and (Bz)_3_ in Ba(Bz)_3_ (*D*_3_, ^1^A_1_) complex, using Ba (S, 6s^0^6p^0^5d^2^) + (Bz)_3_ (S) as interacting complexes (Table 4), and eigenvalues |ν_n_| of the charge flow. The color code of the charge flow is red → blue. The isosurface values are 0.002 for Δ*ρ*_(1)_, 0.0005 for Δ*ρ*_(2)‐(4)_ and 0.0004 for Δ*ρ*_(5)‐(7)_.

The assignment of the Δ*E*_orb(1)_ interaction to (Bz)_3_←M(d), which has a_1_ symmetry, may be questioned because the MO diagram shows that the metal (n)s AO correlates in a *D*_3_ environment also with a_1_ symmetric MOs (Figure 6 b). Inspection of the related deformation density Δ*ρ*_1_ and the associated orbitals reveals that the contribution of the metal (n)s AO to the 2a_1_ MO is negligible. Also, the EDA‐NOCV calculation of the *D*_3*h*_ structures of M(Bz)_3_ gives values that are very similar to the *D*_3_ analysis. The numerical results of the EDA‐NOCV calculation using the *D*_3*h*_ structures are shown in Table S3. Figure 7 displays the shape of the deformation densities Δ*ρ*_1_−Δ*ρ*_7_ of Ba(Bz)_3_ which nicely illustrate the charge alteration due to the pairwise orbital interactions Δ*E*_orb(1)_−Δ*E*_orb(7)_. The color code of the charge flow is red → blue. The deformation densities Δ*ρ*_1_−Δ*ρ*_7_ of M(Bz)_3_ calculated with *D*_3*h*_ symmetry are displayed in Figures S7–S9. The shape of Δ*ρ*_7_ clearly reveals that the small stabilization energy Δ*E*_orb(7)_ is solely due to the polarization of the ligand orbitals. The deformation densities Δ*ρ*_1_−Δ*ρ*_7_ of Ca(Bz)_3_ and Sr(Bz)_3_ closely resemble those of Ba(Bz)_3_. They are shown in Figures S10 and S11.

One referee pointed out that the choice of the electronic reference states of the fragments is somewhat arbitrary and that the same state can be obtained from a ground state metal atom and excited benzene molecules, which would yield a different pattern of donation and back‐donation. This is a justified objection, which needs to be addressed. The electronic ground state of the metal atoms has the electron configuration (n)s^2^, which transforms to a_1_′ in *D*_3*h*_ symmetry. But the shape of the occupied 1a_1_′ MO of the complex Ba(Bz)_3_ shown in Figure 6 a reveals that the 6s AO of Ba has a negligible coefficient in the molecular orbital of the complex, which has only slightly distorted ligand orbitals. The same situation is found in the *D*_3_ symmetric structure (Figure 6 b). The visual impression is supported by EDA‐NOCV calculations of the complexes M(Bz)_3_ using the atoms M in the electronic ground state configuration (n)s^2^ and (Bz)_3_ in the doubly excited singlet state with two electrons in the π* MO. It has been shown by us in numerous studies that those fragments, which give the smallest absolute values for the orbital term Δ*E*_orb_, are the most suitable species to represent the bonding of the complex, since they change the least in the formation of the bond. This method has proven to be very helpful for a large number of chemical compounds whose nature is not directly apparent.^27^, ^28^, ^29^, ^30^, ^31^, ^32^, ^33^, ^34^, ^35^, ^36^, ^37^ Tables S4–S6 show that the Δ*E*_orb_ values using atom M in the electronic ground state and (Bz)_3_ in the excited state are significantly larger than those in Table 4.

However, there is an alternative option for the choice of the interacting fragments. The calculated partial charges of M(Bz)_3_ using the NBO 6.0 method^38^ suggest large positive charges for the metal atoms of 1.47 for Ca, 1.46 for Sr and 1.40 for Ba. The choice of neutral fragments in the electronic references states for M(Bz)_3_ follows the Dewar–Chatt–Duncanson (DCD) model for transition metal compounds, which considers the metal atom and the ligands prior to bond formation.^39^ But the formation of chemical bonds can lead to large charge transfers, resulting in an electronic structure of the molecule that differs greatly from the initial components.

We carried out EDA‐NOCV calculations using charged fragments as interacting moieties. We found that the lowest Δ*E*_orb_ value is given when the singly charged species M^+^ in the doublet state with the electron configuration (n−1)d^1^ns^0^np^0^ and doublet (Bz)_3_^−^ are employed as interacting fragments. Fragments with other charges or electron configurations give larger Δ*E*_orb_ values (see the numerical results in Tables S4–S6 in Supporting Information). Table 5 shows the numerical EDA‐NOCV results for the M^+^‐(Bz)_3_^−^ interactions.

**Table 5:** Results of EDA‐NOCV calculations at BP86‐D3(BJ)/TZ2P for M(Bz)_3_ (*D*_3_, ^1^A_1_) complexes using positively charged atoms M^+^ in the doublet state with ns^0^np^0^(n−1)d^1^ electron configuration and negatively charged (Bz)_3_^−^ (D) as interacting fragments.

Term	Interaction	Ca^+^ (D, 4s^0^4p^0^3d^1^) + (Bz)_3_^−^ (D)	Sr^+^ (D, 5s^0^5p^0^4d^1^) + (Bz)_3_^−^ (D)	Ba^+^ (D, 6s^0^6p^0^5d^1^) + (Bz)_3_^−^ (D)
Δ*E*_int_		−232.9	−228.9	−207.0
Δ*E*_Pauli_		71.4	85.6	110.1
Δ*E*_disp_^[a]^		−7.1 (2.3 %)	−8.4 (2.7 %)	−16.8 (5.3 %)
Δ*E*_elstat_^[a]^		−155.0 (50.9 %)	−159.0 (50.6 %)	−176.7 (55.8 %)
Δ*E*_orb_^[a]^		−142.2 (46.7 %)	−147.0 (46.8 %)	−123.4 (38.9 %)
Δ*E*_orb(1)_^[b]^ (2a_1_)	(Bz)_3_‐M(d) electron‐sharing	−70.9 (49.9 %)	−76.7 (52.2 %)	−53.7 (43.5 %)
Δ*E*_orb(2)_^[b]^ (3e)	(Bz)_3_→M(d) donation	−20.4 (14.3 %)	−22.0 (15.0 %)	−24.2 (19.6 %)
Δ*E*_orb(3)_^[b]^ (2e)	(Bz)_3_→M(d) donation	−19.4 (13.6 %)	−19.4 (13.2 %)	−18.4 (14.9 %)
Δ*E*_orb(4)_^[b]^ (1a_1_)	(Bz)_3_→M(s) donation	−5.5 (3.9 %)	−5.1 (3.5 %)	−4.0 (3.2 %)
Δ*E*_orb(5)_^[b]^ (1a_2_)	(Bz)_3_→M(p) donation	−3.3 (2.3 %)	−2.7 (1.8 %)	−2.4 (1.9 %)
Δ*E*_orb(6)_^[b]^ (1e)	(Bz)_3_→M(p) donation	−6.6 (4.6 %)	−3.0 (2.0 %)	−3.8 (3.1 %)
Δ*E*_orb(7)_^[b]^ (1a_2_)	Polarization	−2.6 (1.8 %)	−3.0 (2.0 %)	−4.3 (3.5 %)
Δ*E*_orb(rest)_^[b]^		−13.5 (9.5 %)	−15.1 (10.3 %)	−12.6 (10.2 %)

[a] The values in parentheses give the percentage contribution to the total attractive interactions Δ*E*_elstat_ + Δ*E*_orb_ + Δ*E*_disp_. [b] The values in parentheses give the percentage contribution to the total orbital interactions Δ*E*_orb_.

**Figure 8:**
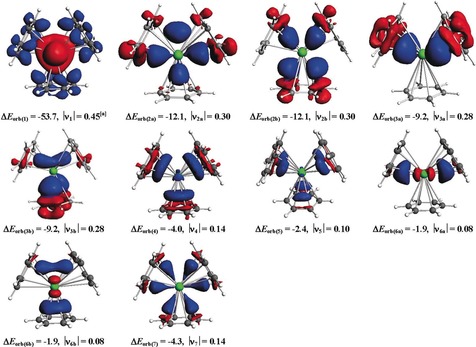
Shape of the deformation densities Δ*ρ*_(1)‐(7)_, which are associated with the orbital interactions Δ*E*_orb(1)‐(7)_ in Ba(Bz)_3_ (*D*_3_, ^1^A_1_) complex, using Ba^+^ (D, 6s^0^6p^0^5d^1^) + (Bz)_3_^−^ (D) as interacting complexes (Table 5), and eigenvalues |ν_n_|of the charge flow. The color code of the charge flow is red → blue. The isosurface values are 0.001 for Δ*ρ*_(1),_ 0.0005 for Δ*ρ*_(2)‐(4)_and 0.0004 for Δ*ρ*_(5)‐(7)_. ^a^Net charge flow of the electron‐sharing interactions.

The data in Table 5 show that the covalent term Δ*E*_orb_ is now a bit smaller than the Coulombic attraction Δ*E*_elstat_. The strongest pairwise orbital interaction Δ*E*_orb(1)_ between the fragments in the electronic doublet state comes from the electron‐sharing bond formation between the unpaired electrons of M^+^ and (Bz)_3_^−^. It is interesting to see that the associated deformation density Δ*ρ*_1_ shown in Figure 8 exhibits a very similar shape as the dative interaction of Δ*E*_orb(1)_ when neutral fragments are used (Figure 7). It means that the electron‐sharing interactions between the (n−1)d electron of M^+^ and the unpaired electron of (Bz)_3_^−^ have a polarity from the metal towards the ligands. As can be expected, the associated eigenvalue υ_1_, which indicates the magnitude of the net charge flow, is much smaller for the charged fragments than for the neutral fragments. The relative contribution of the 2a_1_ orbital interaction between the charged fragments is smaller than the dominating 2a_1_ term of the neutral fragments, whereas the 2e and 3e orbital terms that come from (Bz)_3_→M(d) π donation are larger when charged fragment are used. But the most relevant results is the finding that the (n−1)d AOs of the metal are still clearly the most important valence orbitals for covalent metal‐(Bz)_3_ binding even when charged fragments are used for the EDA‐NOCV calculations.

The two sets of EDA‐NOCV results in Tables 4 and 5 address two different questions. The dissociation of M(Bz)_3_ gives neutral atoms M and three neutral ligands Bz and the data in Table 4 provide information about the total charge flow taking place during bond formation. The latter results directly refer to the DCD model and to orbital correlation diagrams, which always take the orbitals of the fragments prior to bond formation for explaining molecular structures and reactivities.^40^ The data in Table 5 give information about the best description of the bonding situation in the eventually formed complexes, which is given in terms of interactions between the metal ion M^+^ and the negatively charged ligand cave (Bz)_3_^−^. EDA‐NOCV calculations using the doubly charged fragments M^2+^ and (Bz)_3_^2−^ give larger Δ*E*_orb_ values (Tables S4–S6, last columns) than singly charged fragments. The results in Tables 4 and 5 demonstrate the variability of the EDA‐NOCV method, which provides quantitative answer to the questions about the binding interactions between fragments prior and after the bond formation. The difference between the two situations is sometimes not recognized, which has led to a misunderstanding of the EDA‐NOCV results by some authors.^41^

Benzene is an excellent ligand in the organometallic chemistry of low‐valent transition metals. Since the first synthesis of bis(benzene)chromium Cr(Bz)_2_,^18^ a large number of sandwich or half‐sandwich transition metal compounds involving one or two benzene ligands have been reported.^42^ Although main group metal cation–benzene complexes^43^ as well as organometallic complexes of the alkaline earth cations such as metallocene complexes that feature ionic bonding with no propensity for π backbonding are quite common,^44^, ^45^, ^46^, ^47^, ^48^ sandwich complexes of neutral alkaline earth metals, in particular with three η^6^‐bound benzene ligands, are unprecedented. In the literature, only very few metallocene complexes of actinides involving three η^5^‐bound cyclopendadienyl ligands have been reported.^49^ The successful synthesis of trisbenzene complexes of heavier alkaline earth metals may open the door to another chapter of metal complexes.

**Figure 9:**
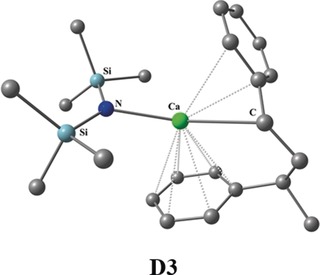
Calculated structure **D3** of a calcium compound sandwiched between two aromatic rings, which is relevant in the hydrogenation of alkene catalyzed by alkaline earth atoms. The Figure is adapted from reference 4.

The observation of the alkaline earth trisbenzene complexes M(Bz)_3_ is not just a surprising finding of some exotic species. It seems that the bonding situation of the arene complexes plays also an important role in some intermediates of alkaline earth catalyzed reactions. Alonso, Harder and co‐workers recently reported the alkene hydrogenation catalyzed by simple compounds of the alkaline earth elements Ca, Sr, Ba.^4^ The calculated reaction profile identified a species **D3** where Ca is sandwiched between two aromatic rings (Figure 9). The structure of **D3** closely resembles the metal–ligand bonds in M(Bz)_3_. A similar situation was reported by the group for the alkaline earth catalyzed imine hydrogenation.^5^ The involvement of the valence‐d orbitals of Ca may also be involved in the recently reported organocalcium‐mediated nucleophilic alkylation of benzene.^3^ The transition‐metal like chemical reactivity of Ca, Ba, Sr is perhaps more relevant for synthetic chemistry than hitherto thought.

## Conclusion

In summary, we report the synthesis and spectroscopic identification of the unprecedented trisbenzene complexes M(Bz)_3_ (M=Sr, Ba) featuring three equivalent η^6^‐bound benzene ligands in low‐temperature Ne matrix. The analysis of the electronic structure shows that the complexes exhibit metal–ligand bonds that are typical for transition metal compounds. The chemical bonds can be explained in terms of weak donation from the π MOs of benzene ligands into the vacant (n−1)d AOs of M and strong backdonation from the occupied (n−1)d AO of M into vacant π* MOs of benzene ligands. The metals in these 20‐electron complexes have 18 effective valence electrons, and thus still fulfill the 18‐electron rule, because two valence electrons occupy a ligand‐based MO that has zero coefficient at the metal. The results suggest that the heavier earth alkaline atoms Ca, Sr, Ba may exhibit the full bonding scenario of transition metals.

## Conflict of interest

The authors declare no conflict of interest.

## Supporting information

As a service to our authors and readers, this journal provides supporting information supplied by the authors. Such materials are peer reviewed and may be re‐organized for online delivery, but are not copy‐edited or typeset. Technical support issues arising from supporting information (other than missing files) should be addressed to the authors.

Supplementary
