# Supplementary material for: Transition‐Metal Chemistry of Alkaline‐Earth Elements: The Trisbenzene Complexes M(Bz)3 (M=Sr, Ba)
Source: Angew Chem Int Ed Engl. 2019 Oct 17;58(48):17365–74. doi: 10.1002/anie.201908572 (PMC6900055; doi:10.1002/anie.201908572)
Supplement: Supplementary file 1 — Supplementary [file ANIE-58-17365-s001.pdf]

Angewandte Chemie
Eine Zeitschrift der Gesellschaft Deutscher Chemiker

Supporting Information

Transition-Metal Chemistry of Alkaline-Earth Elements: The Trisbenzene Complexes $M(Bz)_3$ ($M = Sr$, Ba)

Qian Wang[+], Sudip Pan[+], Yan-Bo Wu[+], Guohai Deng, Jian-Hong Bian, Guanjun Wang, Lili Zhao, Mingfei Zhou, and Gernot Frenking**

anie_201908572_sm_miscellaneous_information.pdf

Supporting Information

Experimental and Computational Details.

Figures S1 – S11.

Tables S1 - S7.

Experimental Details

The 1064 nm fundamental output of a Nd:YAG laser (Continuum, Minilite II; 10 Hz repetition rate) was used to evaporate alkaline earth metal atoms from a rotating metal target, which were co-deposited with benzene molecules in excess neon onto a cryogenic CsI window maintained at 4 K by means of a closed-cycle helium refrigerator. Benzene/Ne mixtures were prepared in a stainless steel vacuum line using a standard manometric technique. C_6H_6 (Sinopharm Chemical Reagwnt Co., Ltd, 99.5%) and isotopic-labeled $^{13}C_6H_6$ (Cambridge isotope laboratories Inc, 99%) and C_6D_6 (Cambridge isotope laboratories Inc, 99.5%) samples were used. After 30 min of sample deposition at 4 K, infrared absorption spectrum was recorded in the transmission mode between 4000 and 450 cm^{-1} using a Bruker Vertex 80V spectrometer at a resolution of 0.5 cm^{-1}. A liquid nitrogen cooled broad band HgCdTe (MCT) detector was used. Bare window backgrounds, recorded prior to sample deposition, were used as references in processing the sample spectra. After the infrared spectrum of the initial deposition had been recorded, the samples were annealed to the desired temperature and quickly re-cooled and more spectra were taken. Broad-band photoexcitation was performed using a high pressure mercury arc lamp with glass filters.

Computational Details

The geometrical optimizations followed by harmonic vibrational frequency computations for $M(Bz)_3$ and $M(Bz)_2$ (M = Ca, Sr, Ba) complexes were done at the M06-2X-D3[1]/def2-TZVPP[2] level. This basis set uses quasi-relativistic effective core potentials for 28 and 46 core electrons for Sr and Ba atoms, respectively. All these computations were carried out using the Gaussian 16 program package.[3] Superfine integration grid was used for the computations.

The bonding situation was studied via energy decomposition analysis (EDA)[4] together with the natural orbitals for chemical valence (NOCV)[5] method by using the ADF 2017.01 program package.[6] The EDA-NOCV[7] calculations were performed at the BP86-D3(BJ)/TZ2P[8]level where the scalar relativistic effects were included by adopting the zeroth-order regular approximation (ZORA).[9] In the EDA method, the intrinsic interaction energy (ΔE_{int}) between two fragments is decomposed into four energy components (eq. 1).

$$\Delta E_{\text{int}} = \Delta E_{\text{elstat}} + \Delta E_{\text{Pauli}} + \Delta E_{\text{disp}} + \Delta E_{\text{orb}} \quad (1).$$

The ΔE_{elstat} term represents the quasiclassical electrostatic interaction between the unperturbed charge distributions of the prepared fragments. The Pauli repulsion ΔE_{Pauli} is the energy change associated with the transformation from the superposition of the unperturbed electron densities of the isolated fragments to the wave function, which properly obeys the Pauli principle through explicit antisymmetrization and renormalization of the product wave function. The ΔE_{disp} term corresponds to the dispersion interaction involved in between the fragments. The term ΔE_{orb} is originated from the mixing of orbitals, charge transfer and polarization between the isolated fragments.

The combination of EDA with NOCV method allows us to partition the total ΔE_{orb} term into pairwise contributions of the orbital interactions. The electron density deformation $\Delta\rho_k(r)$, which is originated from the mixing of the orbital pairs $\psi_{\text{k}}(r)$ and $\psi_{\text{-k}}(r)$ of the interacting fragments in the complex, represents the amount and the shape of the charge flow due to the orbital interactions (eq. 2), whereas the associated orbital energy term reflects the strength of such orbital interactions (eq. 3).

$$\Delta\rho_{orb}(r) = \sum_k \Delta\rho_k(r) = \sum_{k=1}^{N/2} \nu_k[-\psi_{-k}^2(r) + \psi_k^2(r)] \quad (2)$$

$$\Delta E_{orb} = \sum_k \Delta E_k^{orb} = \sum_{k=1}^{N/2} \nu_k[-F_{-k,-k}^{TS} + F_{k,k}^{TS}] \quad (3)$$

Therefore, both qualitative ($\Delta\rho_{orb}$) and quantitative (ΔE_{orb}) information of the strength of individual pairs of orbital interactions can be obtained from an EDA-NOCV analysis. For further details on the EDA-NOCV method and its application to the analysis of the chemical bond, some recent reviews are recommended.[10]

[1] Y. Zhao, D. G. Truhlar, Theor. Chem. Acc. 2006, 120 , 215–241.

[2] a) F. Weigend, R. Ahlrichs, *Phys. Chem. Chem. Phys*. **2005**, *7*, 3297; b) F. Weigend, *Phys. Chem. Chem. Phys*., **2006**, *8*, 1057.

[3] Gaussian 16, Revision A.03, M. J. Frisch, G. W. Trucks, H. B. Schlegel, G. E. Scuseria, M. A. Robb, J. R. Cheeseman, G. Scalmani, V. Barone, G. A. Petersson, H. Nakatsuji, X. Li, M. Caricato, A. V. Marenich, J. Bloino, B. G. Janesko, R. Gomperts, B. Mennucci, H. P. Hratchian, J. V. Ortiz, A. F. Izmaylov, J. L. Sonnenberg, D. Williams-Young, F. Ding, F. Lipparini, F. Egidi, J. Goings, B. Peng, A. Petrone, T. Henderson, D. Ranasinghe, V. G. Zakrzewski, J. Gao, N. Rega, G. Zheng, W. Liang, M. Hada, M. Ehara, K. Toyota, R. Fukuda, J. Hasegawa, M. Ishida, T. Nakajima, Y. Honda, O. Kitao, H. Nakai, T. Vreven, K. Throssell, J. A. Montgomery, Jr., J. E. Peralta, F. Ogliaro, M. J. Bearpark, J. J. Heyd, E. N. Brothers, K. N. Kudin, V. N. Staroverov, T. A. Keith, R. Kobayashi, J. Normand, K. Raghavachari, A. P. Rendell, J. C. Burant, S. S. Iyengar, J. Tomasi, M. Cossi, J. M. Millam, M. Klene, C. Adamo, R. Cammi, J. W. Ochterski, R. L. Martin, K. Morokuma, O. Farkas, J. B. Foresman, D. J. Fox, Gaussian, Inc., Wallingford CT, 2016.

[4] T. Ziegler, A. Rauk, *Theor. Chim. Acta* **1977**, *46*, 1.

[5] a) M. Mitoraj, A. Michalak, *Organometallics*, **2007**, *26*, 6576; b) M. Mitoraj, A. Michalak, *J. Mol. Model*. **2008**, *14*, 681.

[6] a) ADF2017, SCM, Theoretical Chemistry, Vrije Universiteit, Amsterdam, The Netherlands, http://www.scm.com; b) G. te Velde, F. M. Bickelhaupt, E. J. Baerends, C. F. Guerra, S. J. A. Van Gisbergen, J. G. Snijders, T. Ziegler, *J. Comput. Chem*. **2001**, *22*, 931.

[7] a) A. Michalak, M. Mitoraj, T. Ziegler, *J. Phys. Chem. A* **2008,** *112*, 1933; b) M. P. Mitoraj, A. Michalak, T. Ziegler, *J. Chem. Theory Comput.* **2009**, *5*, 962.

[8] E. van Lenthe, E. J. Baerends, *J. Comput. Chem*. **2003**, *24*, 1142.

[9] E. van Lenthe, A. Ehlers, E. J. Baerends, *J. Chem. Phys*. **1999**, *110*, 8943.

[10] a) L. Zhao, M. von Hopffgarten, D. M. Andrada, G. Frenking, *WIREs Comput. Mol. Sci.*, **2018**, *8*, e1345; b) G. Frenking, F. M. Bickelhaupt, *The EDA Perspective of Chemical Bonding* in *The Chemical Bond. Fundamental Aspects of Chemical Bonding*, G. Frenking and S. Shaik (Eds), Wiley-VCH, Weinheim, **2014**, p. 121-158; c) G. Frenking, R. Tonner, S. Klein, N. Takagi, T. Shimizu, A. Krapp, K. K. Pandey, P. Parameswaran, *Chem. Soc. Rev*. **2014**, *43*, 5106; d) G. Frenking, F. M. Bickelhaupt, The EDA Perspective of

Chemical Bonding. In The Chemical Bond 1. Fundamental Aspects of Chemical Bonding, G. Frenking, S. Shaik, Eds. Wiley-VCH: Weinheim, **2014** pp 121- 158; e) L. Zhao, M. Hermann, N. Holzmann, G. Frenking, *Coord. Chem. Rev*. **2017**, *344*, 163; f) G. Frenking, M. Hermann, D. M. Andrada, N. Holzmann, *Chem. Soc. Rev*. **2016**, *45*, 1129.

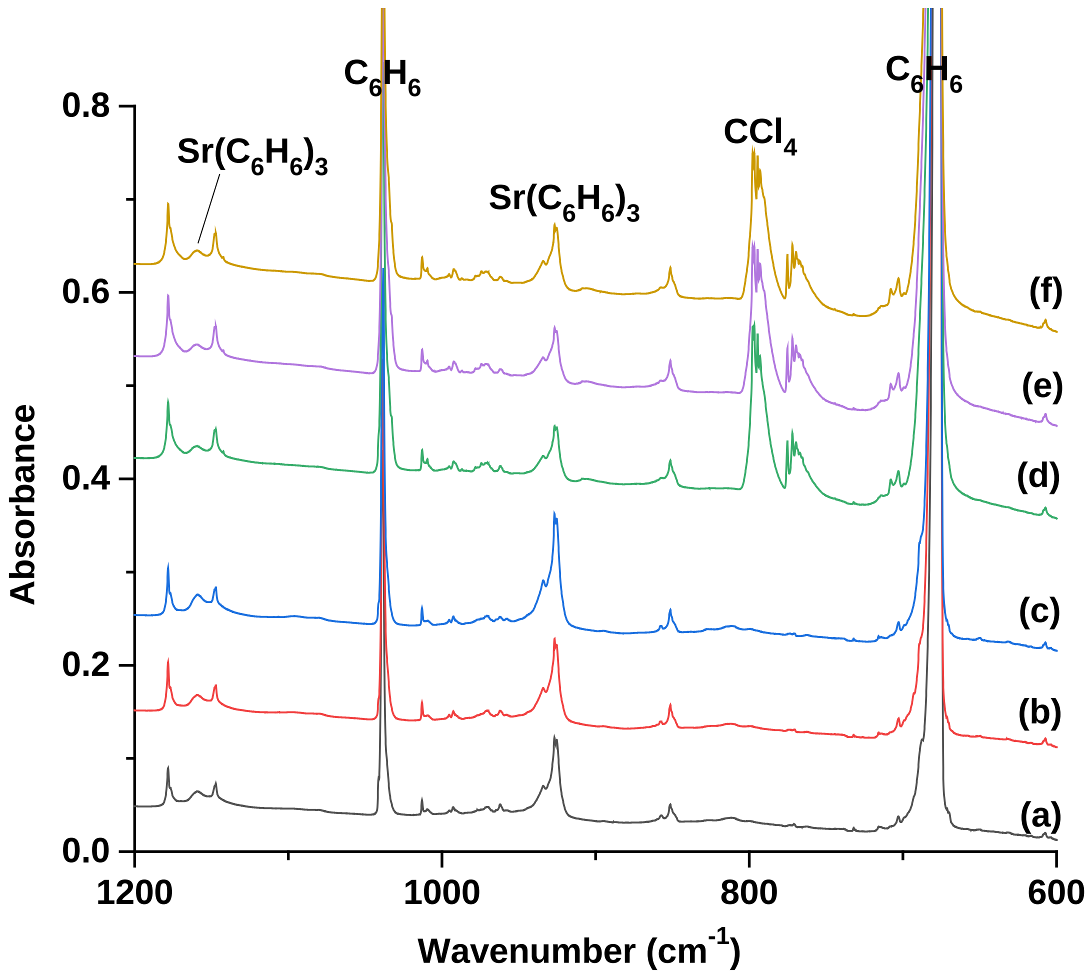

Figure S1. Infrared absorption spectra of strontium-benzene complexes in the 1200-600 cm^{-1} region. a)-c) 0.1% C_6H_6 in neon. a) 30 min of sample deposition at 4 K, b) after annealing at 12 K, c) after 20 min of visible light irradiation, d)-f) 0.1% C_6H_6 + 0.01% CCl_4 in neon d) 30 min of sample deposition at 4 K, e) after annealing at 12 K, and f) after 20 min of visible light irradiation.

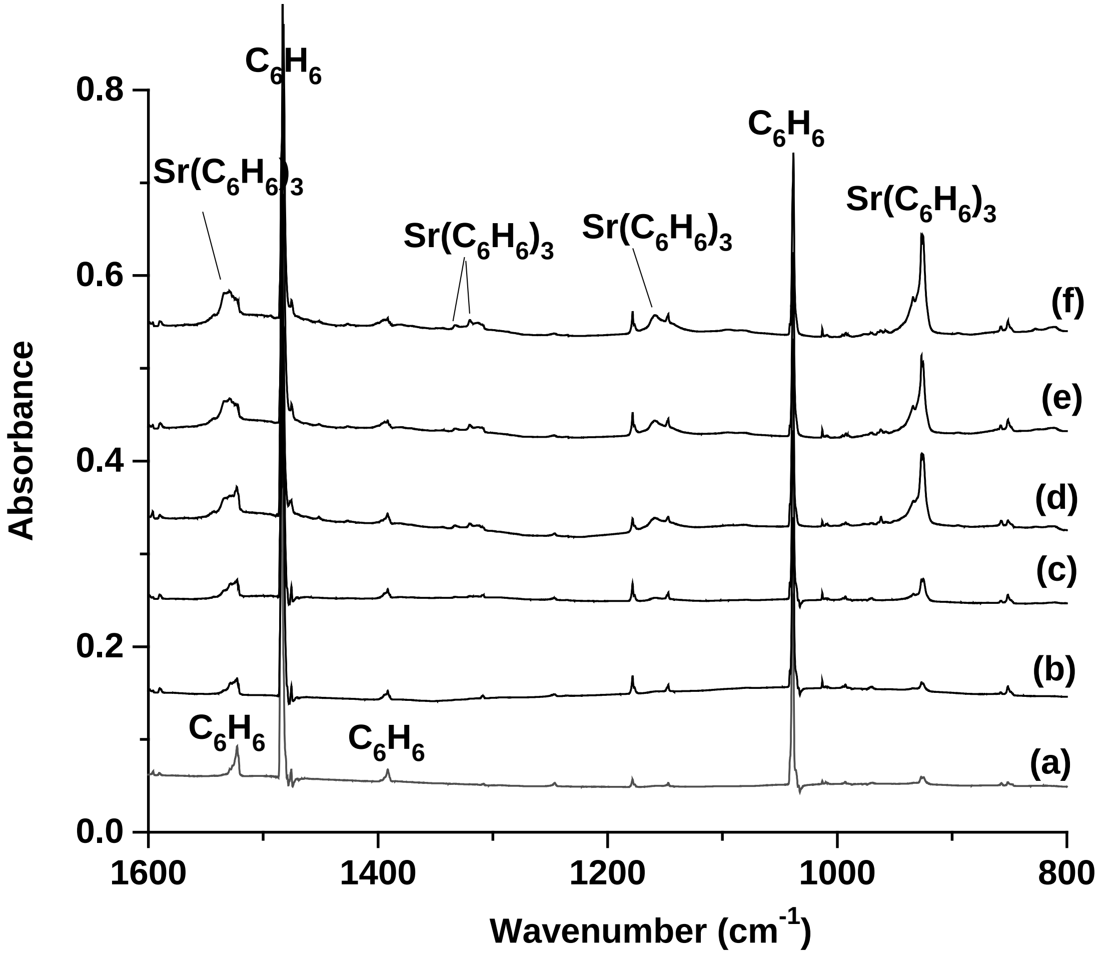

Figure S2. Infrared absorption spectra of strontium-benzene complexes in the 1600-600 cm^{-1} region from co-deposition of laser-evaporated strontium atoms with 0.1% C_6H_6 in neon. a)-c) low laser energy, a) 30 min of sample deposition at 4 K, b) after annealing at 12 K, c) after 20 min of visible light irradiation, d)-f) relatively high laser energy, d) 30 min of sample deposition at 4 K, e) after annealing at 12 K, and f) after 20 min of visible light irradiation.

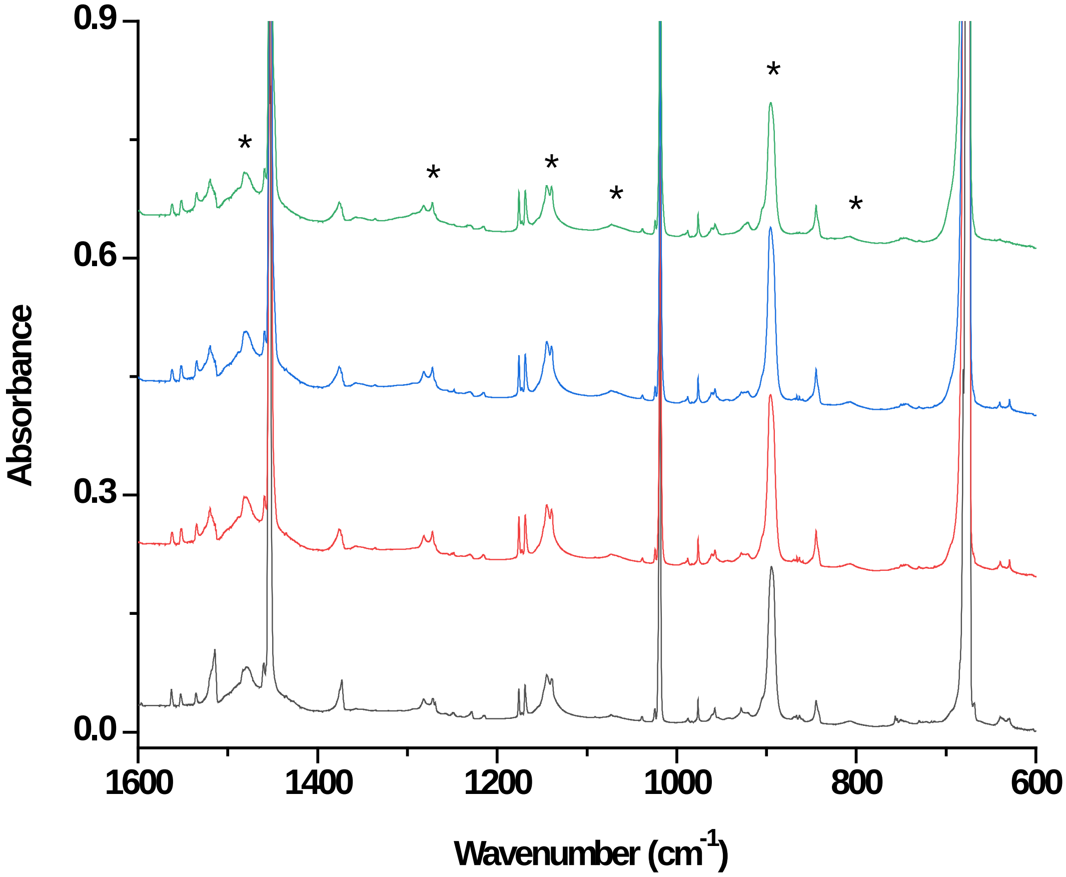

Figure S3. Infrared absorption spectra of barium-benzene complexes in the 1600-600 cm^{-1} region from co-deposition of laser-evaporated barium atoms with 0.2% $^{13}C_6H_6$ in neon. a) 30 min of sample deposition at 4 K, b) after annealing at 12 K, c) after 20 min of visible light irradiation, and (d) after 20 min UV-visible light irradiation. *denotes absorptions of $Ba(^{13}C_6H_6)_3$.

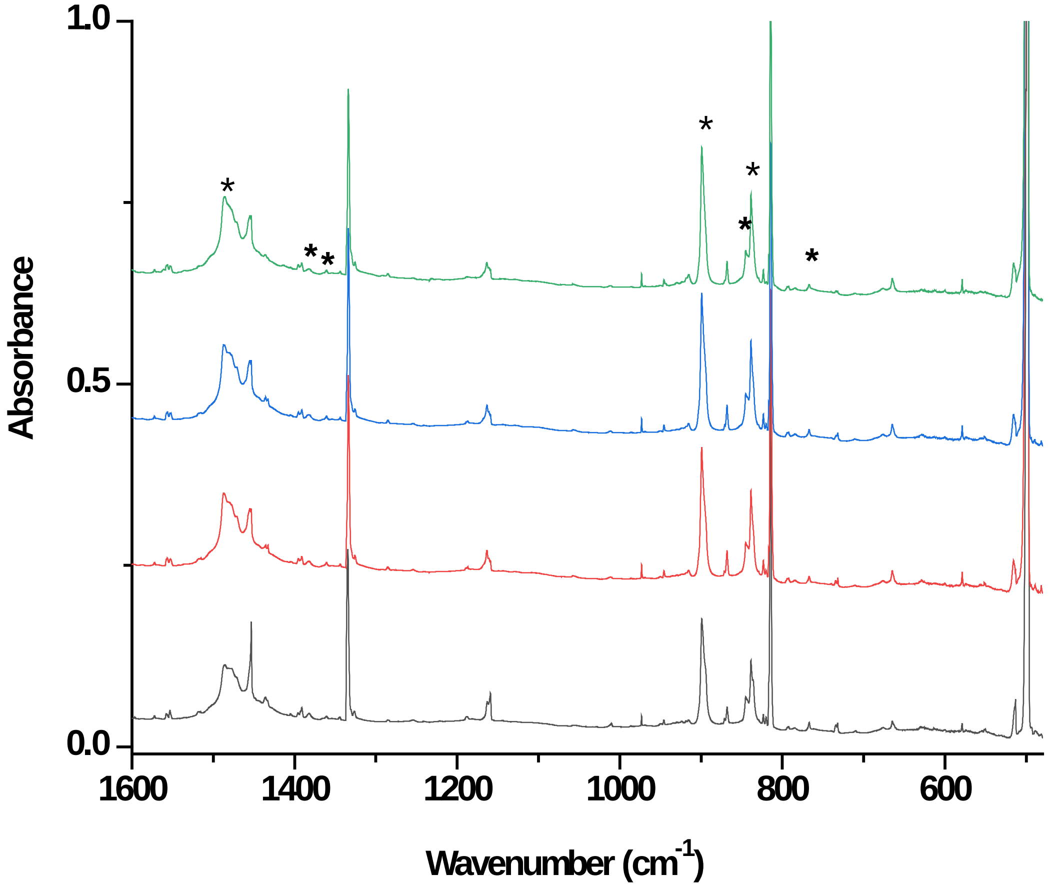

Figure S4. Infrared absorption spectra of barium-benzene complexes in the 1600-600 cm^{-1} region from co-deposition of laser-evaporated barium atoms with 0.2% C_6D_6 in neon. a) 30 min of sample deposition at 4 K, b) after annealing at 12 K, c) after 20 min of visible light irradiation, and (d) after 20 min UV-visible light irradiation. * denotes absorptions of $Ba(C_6D_6)_3$.

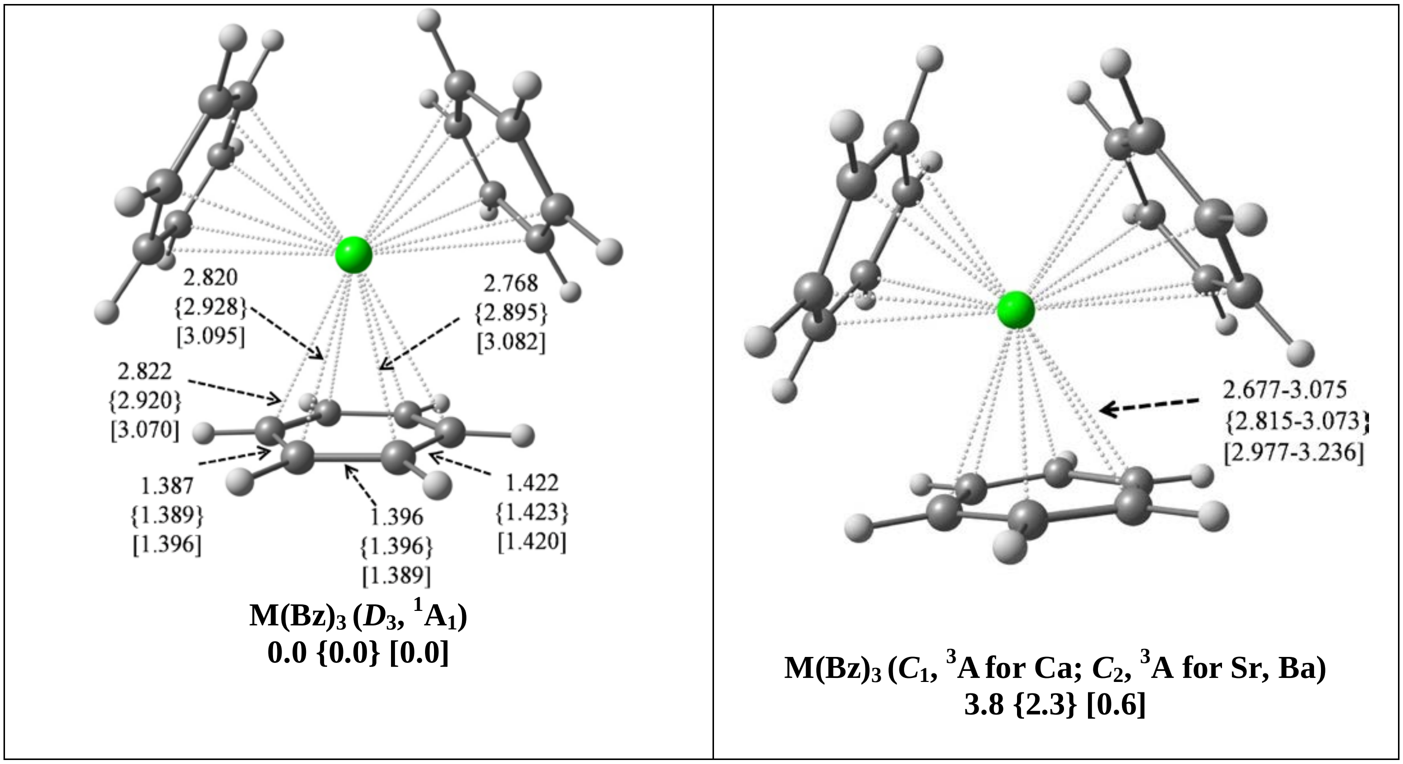

Figure S5 M06-2X-D3/def2-TZVPP geometries of $M(Bz)_3$ complexes M = Ca, {Sr}, [Ba] in singlet and triplet spin states. Bond distances are in Å. ZPE corrected relative energies are in kcal/mol. C_2 symmetric triplet $Ca(Bz)_3$ complex has one imaginary frequency (24.9 *i*).

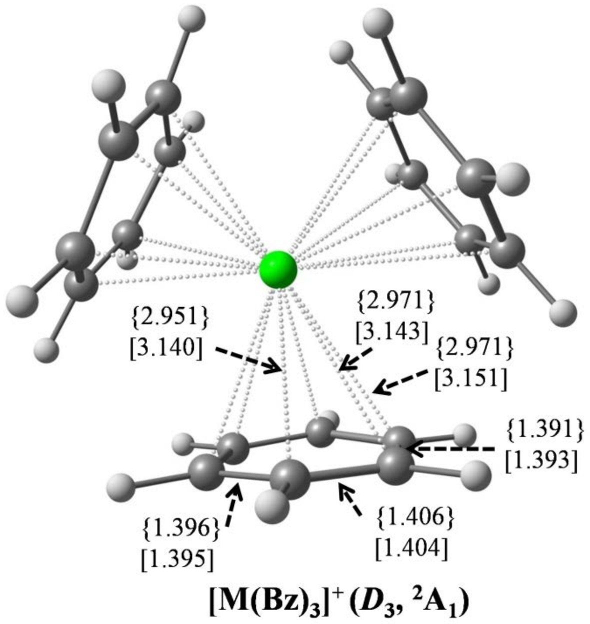

Figure S6. Calculated geometries of the $[M(Bz)_3]^+$ complexes (M = {Sr}, [Ba]) at M06-2X-D3/def2-TZVPP. Bond distances are in Å.

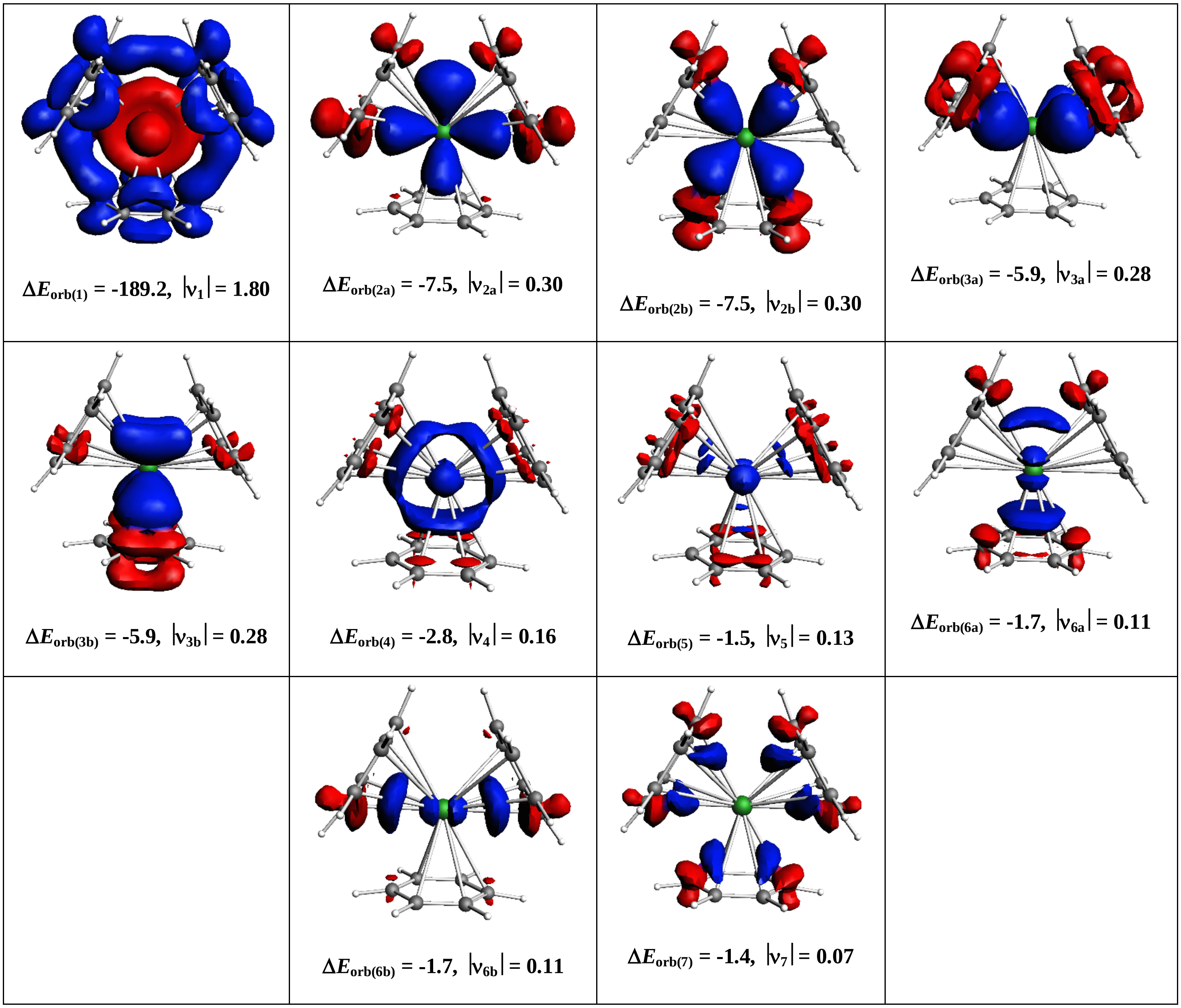

Figure S7. Shape of the deformation densities $\Delta\rho_{(1)\text{-}(7)}$, which are associated with the orbital interactions $\Delta E_{orb(1)\text{-}(7)}$ in Ca(Bz)$_3$ (D_{3h}, $^1A_1'$) complex and eigenvalues $|\nu_n|$ of the charge flow.The color code of the charge flow is red → blue. The isosurface values are 0.002 for $\Delta\rho_{(1)}$, 0.0005 for $\Delta\rho_{(2)\text{-}(6)}$and 0.0003 for $\Delta\rho_{(7)}$.

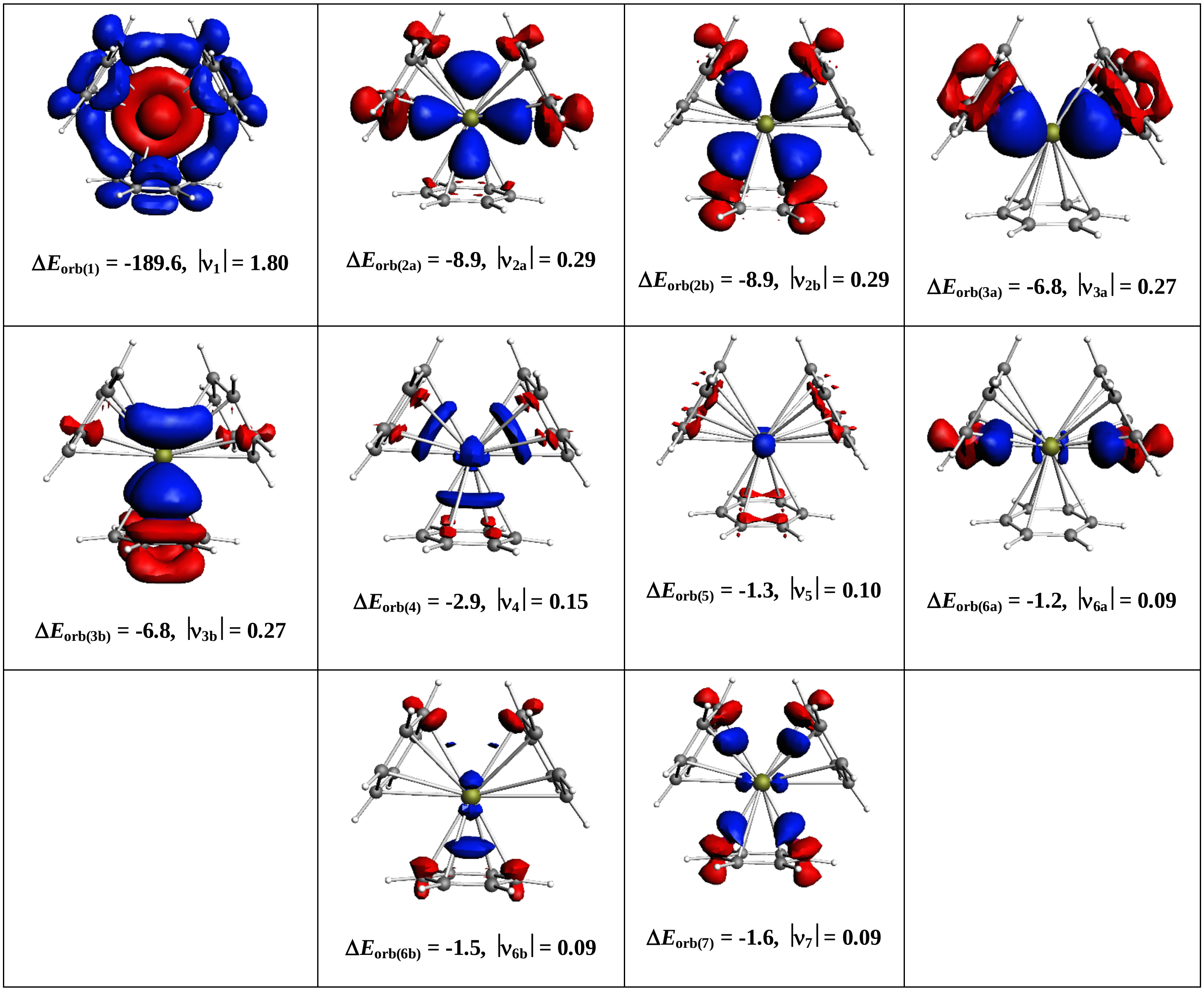

Figure S8. Shape of the deformation densities $\Delta\rho_{(1)\text{-}(7)}$, which are associated with the orbital interactions $\Delta E_{orb(1)\text{-}(7)}$ in Sr(Bz)$_3$ (D_{3h}, $^1A_1'$) complex and eigenvalues $|\nu_n|$ of the charge flow.The color code of the charge flow is red → blue. The isosurface values are 0.002 for $\Delta\rho_{(1),}$ 0.0005 for $\Delta\rho_{(2)\text{-}(6)}$and 0.0003 for $\Delta\rho_{(7)}$.

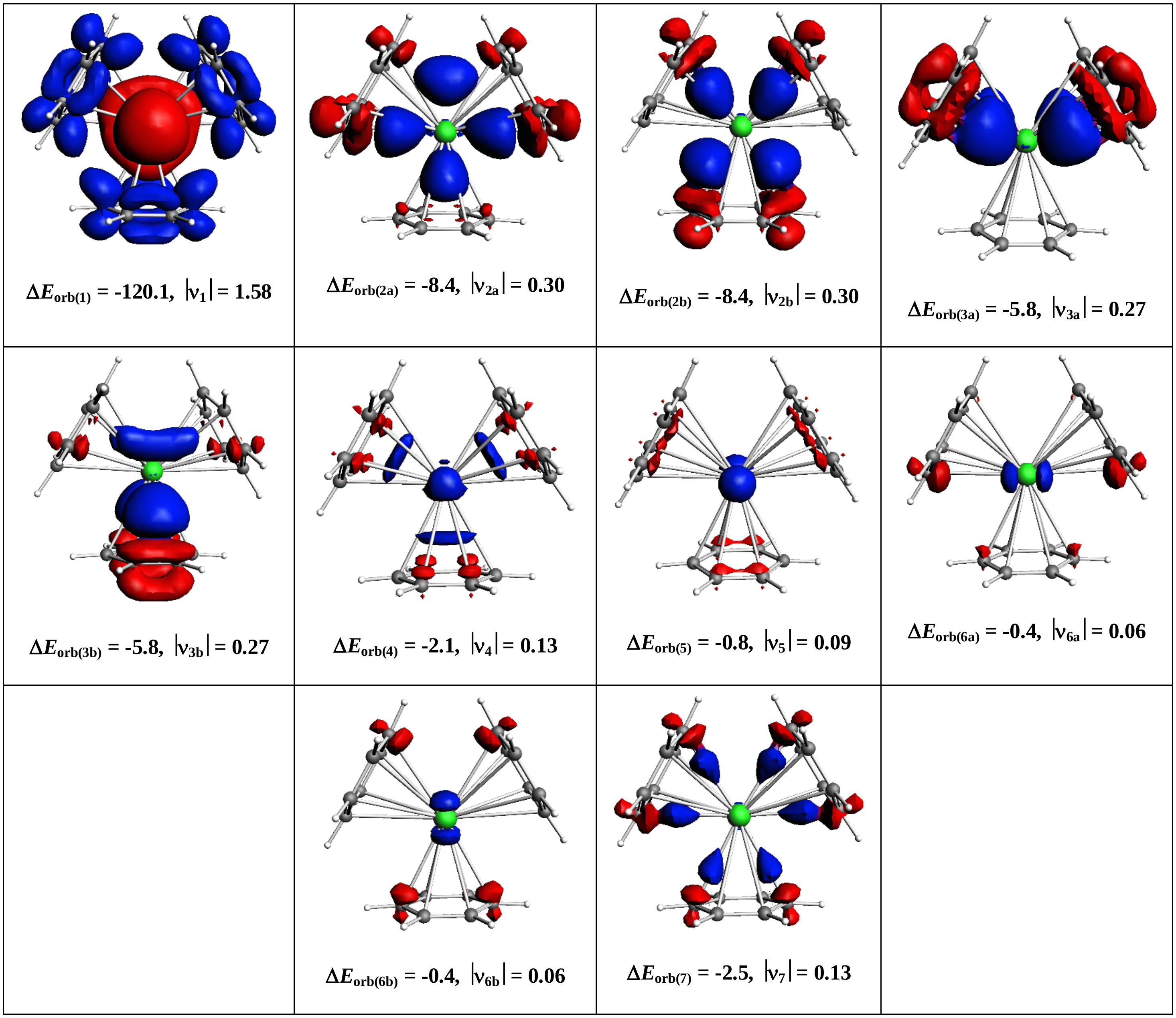

Figure S9. Shape of the deformation densities $\Delta\rho_{(1)\text{-}(7)}$, which are associated with the orbital interactions $\Delta E_{orb(1)\text{-}(7)}$ in $Ba(Bz)_3$ (D_{3h}, $^1A_1'$) complex and eigenvalues $|\nu_n|$ of the charge flow.The color code of the charge flow is red → blue. The isosurface values are 0.002 for $\Delta\rho_{(1)}$, 0.0005 for $\Delta\rho_{(2)\text{-}(4)}$and 0.0004 for $\Delta\rho_{(5)\text{-}(7)}$.

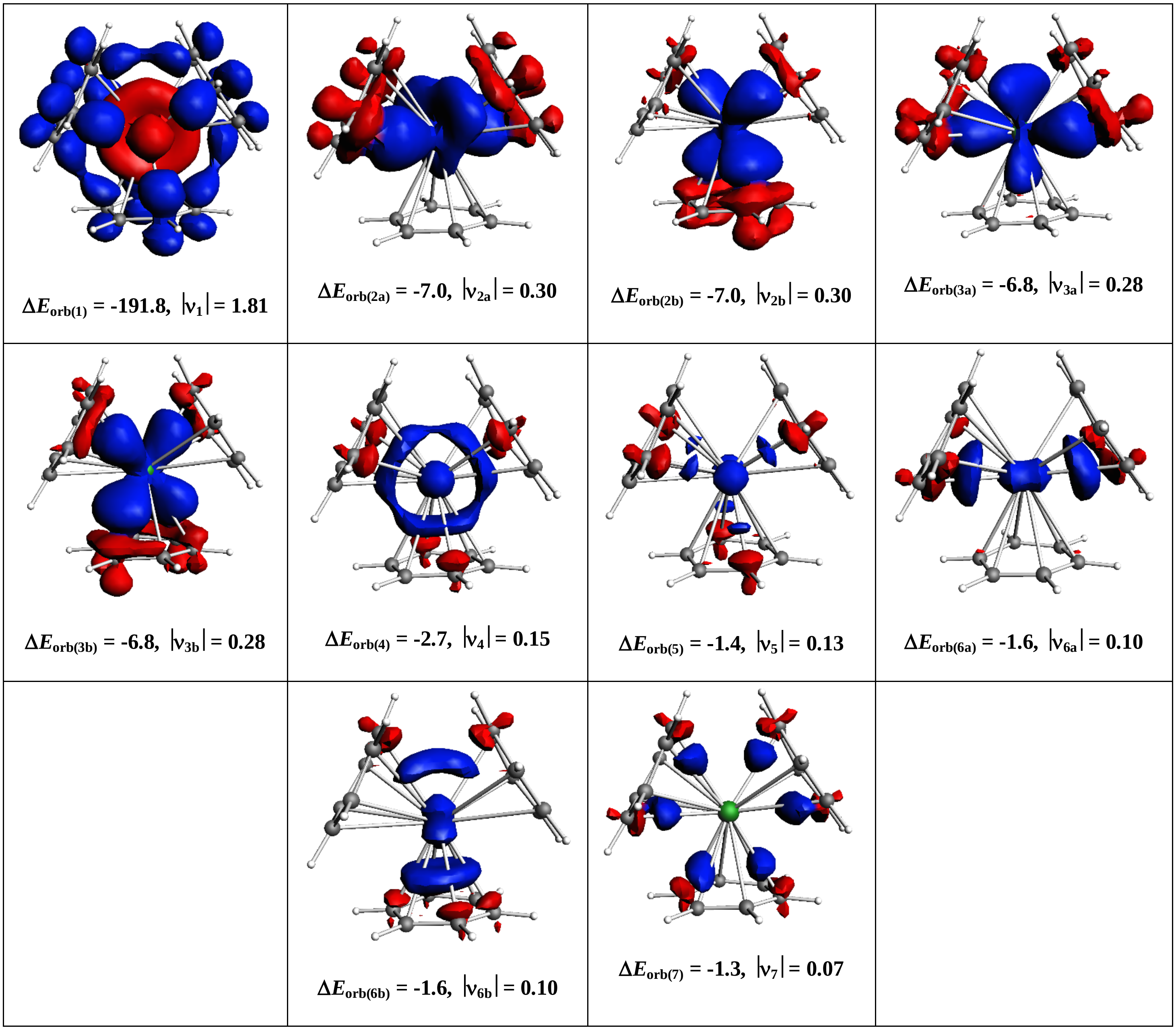

Figure S10. Shape of the deformation densities $\Delta\rho_{(1)\text{-}(7)}$, which are associated with the orbital interactions $\Delta E_{orb(1)\text{-}(7)}$ in $Ca(Bz)_3$ (D_3, $^1A_1$) complex and eigenvalues $|\nu_n|$ of the charge flow.The color code of the charge flow is red → blue. The isosurface values are 0.002 for $\Delta\rho_{(1),}$ 0.0005 for $\Delta\rho_{(2)\text{-}(6)}$and 0.0003 for $\Delta\rho_{(7)}$.

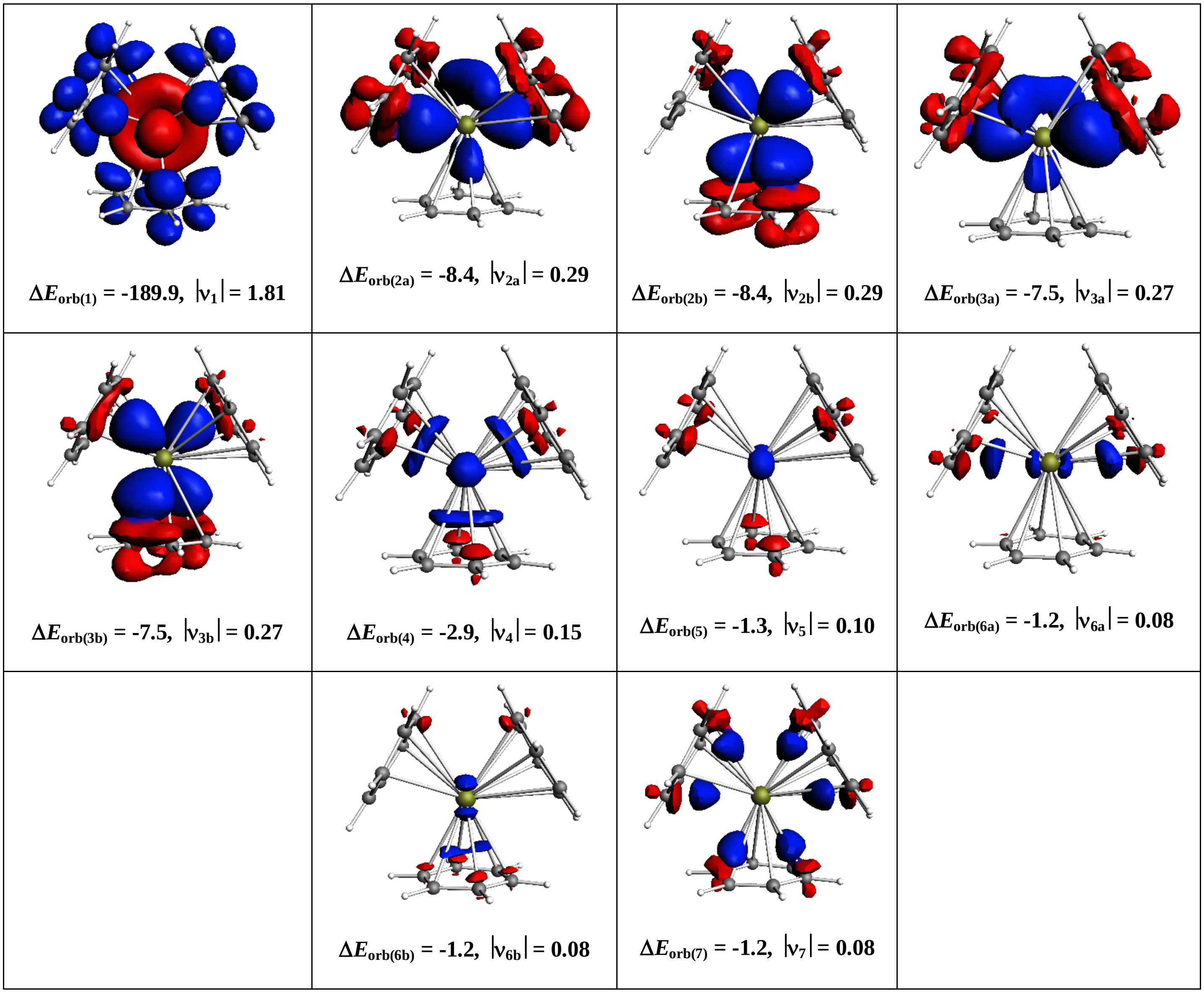

Figure S11. Shape of the deformation densities $\Delta\rho_{(1)\text{-}(7)}$, which are associated with the orbital interactions $\Delta E_{orb(1)\text{-}(7)}$ in $Sr(Bz)_3$ (D_3, $^1A_1$) complex and eigenvalues $|\nu_n|$ of the charge flow. The color code of the charge flow is red → blue. The isosurface values are 0.002 for $\Delta\rho_{(1)}$, 0.0005 for $\Delta\rho_{(2)\text{-}(6)}$ and 0.0003 for $\Delta\rho_{(7)}$.

Table S1. Calculated infrared absorptions (cm^{-1}) of $[Sr(C_6H_6)_3]^+$, $[Sr(^{13}C_6H_6)_3]^+$ and $[Sr(C_6D_6)_3]^+$ at the M06-2X-D3/def2-TZVPP level (D_3 symmetry). Calculated IR intensities (km/mol) are given in parentheses.

Mode		Calculated				
		$^{12}C_6H_6$	$^{13}C_6H_6$	Δ^a	$^{12}C_6D_6$	Δ^a
C=C stretch		1592.8 (307)	1540.4 (263)	-52.4	1542.8 (374)	-50.0
C=C stretch		1580.9 (216)	1529.6 (195)	-51.3	1530.4 (214)	-50.5
C=C stretch and C-D in-plane bend					1366.1 (12)	
C=C stretch and C-D in-plane bend					1353.0 (4)	
C=C stretch and C-H in-plane bend		1337.8 (16)	1291.2 (13)	-46.6	1314.6 (14)	-23.2
C=C stretch and C-H in-plane bend		1329.8 (13)	1284.3 (11)	-45.5	1310.3 (11)	-19.5
C-H(D) in-plane bend		1188.3 (88)	1179.7 (107)	-8.6	872.4 (20)	-315.9
C-H(D) in-plane bend		1180.0 (10)	1166.1 (13)	-13.9	866.3 (21)	-313.7
C-H(D) out-of-plane bend		956.8 (132)	943.9 (183)	-12.9	826.4 (24)	-130.4
Ring breath		994.7 (118)	962.8 (42)	-31.9	952.1 (86)	-42.6
C-D out-of-plane bend					775.8 (82)	
C-H(D) out-of-plane bend		896.3 (4)	889.0 (5)	-7.3	700.0 (1)	-196.3
C-H(D) out-of-plane bend		711.3 (21)	708.9 (24)	-2.4	521.3 (6)	-190.0

[a]Shift with respect to $^{12}C_6H_6$.

Table S2. Calculated infrared absorptions (cm^{-1}) of $[Ba(C_6H_6)_3]^+$, $[Ba(^{13}C_6H_6)_3]^+$ and $[Ba(C_6D_6)_3]^+$ at the M06-2X-D3/def2-TZVPP level (D_3 symmetry). Calculated IR intensities (km/mol) are given in parentheses.

Mode		Calculated				
		$^{12}C_6H_6$	$^{13}C_6H_6$	Δ^a	$^{12}C_6D_6$	Δ^a
C=C stretch		1601.6 (263)	1548.6 (224)	-53.0	1552.1 (330)	-49.5
C=C stretch		1589.4 (216)	1537.2 (196)	-52.2	1540.4 (206)	-49.0
C=C stretch and C-D in-plane bend					1364.5 (10)	
C=C stretch and C-D in-plane bend					1354.1 (1)	
C=C stretch and C-H in-plane bend		1334.5 (16)	1288.6 (14)	-45.9	1315.3 (16)	-19.2
C=C stretch and C-H in-plane bend		1326.6 (10)	1281.9 (8)	-44.7	1308.9 (11)	-17.7
C-H(D) in-plane bend		1189.8 (81)	1181.5 (98)	-8.3	873.1 (17)	-316.7
C-H(D) in-plane bend		1182.9 (16)	1175.2 (13)	-7.7	867.7 (21)	-315.2
C-H(D) out-of-plane bend		961.6 (69)	949.3 (116)	-12.3	819.9 (30)	-141.7
Ring breath		996.6 (130)	964.1 (57)	-32.5	953.8 (81)	-42.8
C-D out-of-plane bend					779.5 (36)	
C-H(D) out-of-plane bend		894.5 (4)	887.1 (5)	-7.4	698.6 (1)	-195.9
C-H(D) out-of-plane bend		706.6 (33)	704.4 (33)	-2.2	518.2 (10)	-188.4

[a]Shift with respect to $^{12}C_6H_6$.

Table S3. The results of EDA-NOCV for $M(Bz)_3$ (D_{3h}, $^1A_1'$) complexes using M (S, $ns^0np^0(n-1)d^2$) as one fragment and $(Bz)_3$ (S) as another fragment at the BP86-D3(BJ)/TZ2P-ZORA//BP86-D3(BJ)/def2-TZVPP level.

Energies	Interaction	Ca (S, $4s^04p^03d^2$) + $(Bz)_3$ (S)	Sr (S, $5s^05p^04d^2$) + $(Bz)_3$ (S)	Ba (S, $6s^06p^05d^2$) + $(Bz)_3$ (S)
ΔE_{int}		-196.5	-192.5	-136.2
ΔE_{Pauli}		120.1	137.4	156.0
ΔE_{disp}[a]		-7.1 (2.2%)	-8.5 (2.6%)	-16.8 (5.7%)
ΔE_{elstat}[a]		-80.9 (25.6%)	-87.7 (26.6%)	-116.3 (39.8%)
ΔE_{orb}[a]		-228.6 (72.2%)	-233.7 (70.8%)	-159.1 (54.4%)
$\Delta E_{orb(1)}$[b] (2a₁’)	$(Bz)_3 \leftarrow M(d)$ backdonation	-189.2 (82.8%)	-189.6 (81.1%)	-120.1 (75.5%)
$\Delta E_{orb(2)}$[b] (2e’)	$(Bz)_3 \rightarrow M(d)$ donation	-15.0 (6.6%)	-17.8 (7.6%)	-16.8 (10.6%)
$\Delta E_{orb(3)}$[b] (1e’’)	$(Bz)_3 \rightarrow M(d)$ donation	-11.8 (5.2%)	-13.6 (5.8%)	-11.6 (7.3%)
$\Delta E_{orb(4)}$[b] (1a₁’)	$(Bz)_3 \rightarrow M(s)$ donation	-2.8 (1.2%)	-2.9 (1.2%)	-2.1 (1.3%)
$\Delta E_{orb(5)}$[b] (1a₂’’)	$(Bz)_3 \rightarrow M(p)$ donation	-1.5 (0.7%)	-1.3 (0.6%)	-0.8 (0.5%)
$\Delta E_{orb(6)}$[b] (1e’)	$(Bz)_3 \rightarrow M(p)$ donation	-3.4 (1.5%)	-2.7 (1.2%)	-0.8 (0.5%)
$\Delta E_{orb(7)}$[b] (1a₂’)	Polarization	-1.4 (0.6%)	-1.6 (0.7%)	-2.5 (1.6%)
$\Delta E_{orb(rest)}$[b]		-3.5 (1.5%)	-4.2 (1.8%)	-4.4 (2.8%)

[a]The values in parentheses give the percentage contribution to the total attractive interactions $\Delta E_{elstat} + \Delta E_{orb} + \Delta E_{disp}$

[b]The values in parentheses give the percentage contribution to the total orbital interactions ΔE_{orb}.

Table S4. The results of EDA-NOCV for $Ca(Bz)_3$ (D_3, $^1A_1$) complexes using different fragmentation schemes at the BP86-D3(BJ)/TZ2P-ZORA level.

Energies	Ca (S, $4s^04p^03d^2$) + $(Bz)_3$ (S)	Ca (T, $4s^04p^03d^2$) + $(Bz)_3$ (T)	Ca (S, $4s^24p^03d^0$) + $(Bz)_3$ (S, Ex)	Ca^+ (D, $4s^04p^03d^1$) + $(Bz)_3^-$ (D)	Ca^+ (D, $4s^14p^03d^0$) + $(Bz)_3^-$ (D, Ex)	Ca^{2+} (S, $4s^04p^03d^0$) + $(Bz)_3^{2-}$ (S)
ΔE_{int}	-197.4	-386.7	-406.9	-232.9	-375.7	-568.6
ΔE_{Pauli}	124.5	101.9	296.2	71.4	142.6	74.2
ΔE_{disp}[a]	-7.1 (2.2%)	-7.1 (1.5%)	-7.1 (1.0%)	-7.1 (2.3%)	-7.1 (1.4%)	-7.1 (1.1%)
ΔE_{elstat}[a]	-82.7 (25.7%)	-85.4 (17.5%)	-267.6 (38.1%)	-155.0 (50.9%)	-239.6 (46.2%)	-435.8 (67.8%)
ΔE_{orb}[a]	-232.1 (72.1%)	-396.1 (81.1%)	-428.4 (60.9%)	-142.2 (46.7%)	-271.7 (52.4%)	-199.9 (31.1%)

[a]The values in parentheses give the percentage contribution to the total attractive interactions $\Delta E_{elstat} + \Delta E_{orb} + \Delta E_{disp}$

Table S5. The results of EDA-NOCV for $Sr(Bz)_3$ (D_3, $^1A_1$) complexes using different fragmentation schemes at the BP86-D3(BJ)/TZ2P-ZORA level.

Energies	Sr (S, $5s^05p^04d^2$) + $(Bz)_3$ (S)	Sr (T, $5s^05p^04d^2$) + $(Bz)_3$ (T)	Sr (S, $5s^25p^04d^0$) + $(Bz)_3$ (S, Ex)	Sr^+ (D, $5s^05p^04d^1$) + $(Bz)_3^-$ (D)	Sr^+ (D, $5s^15p^04d^0$) + $(Bz)_3^-$ (D, Ex)	Sr^{2+} (S, $5s^05p^04d^0$) + $(Bz)_3^{2-}$ (S)
ΔE_{int}	-191.5	-385.5	-398.5	-228.9	-360.5	-535.8
ΔE_{Pauli}	140.5	164.2	305.4	85.6	155.7	87.0
ΔE_{disp}[a]	-8.4 (2.5%)	-8.4 (1.5%)	-8.4 (1.2%)	-8.4 (2.7%)	-8.4 (1.6%)	-8.4 (1.3%)
ΔE_{elstat}[a]	-88.8 (26.8%)	-90.8 (16.5%)	-265.4 (37.7%)	-159.0 (50.6%)	-241.4 (46.8%)	-434.5 (69.8%)
ΔE_{orb}[a]	-234.7 (70.7%)	-450.4 (82.0%)	-430.0 (61.1%)	-147.0 (46.8%)	-266.4 (51.6%)	-179.9 (28.9%)

[a]The values in parentheses give the percentage contribution to the total attractive interactions $\Delta E_{elstat} + \Delta E_{orb} + \Delta E_{disp}$

Table S6. The results of EDA-NOCV for $Ba(Bz)_3$ (D_3, $^1A_1$) complexes using different fragmentation schemes at the BP86-D3(BJ)/TZ2P-ZORA level.

Energies	Ba (S, $6s^0 6p^0 5d^2$) + $(Bz)_3$ (S)	Ba (T, $6s^0 6p^0 5d^2$) + $(Bz)_3$ (T)	Ba (S, $6s^2 6p^0 5d^0$) + $(Bz)_3$ (S, Ex)	Ba^+ (D, $6s^0 6p^0 5d^1$) + $(Bz)_3^-$ (D)	Ba^+ (D, $6s^1 6p^0 5d^0$) + $(Bz)_3^-$ (D, Ex)	Ba^{2+} (S, $6s^0 6p^0 5d^0$) + $(Bz)_3^{2-}$ (S)
ΔE_{int}	-135.5	-322.9	-412.8	-207.0	-365.9	-519.2
ΔE_{Pauli}	156.8	282.2	301.4	110.1	159.4	87.3
ΔE_{disp}[a]	-16.8 (5.7%)	-16.8 (2.7%)	-16.8 (2.5%)	-16.8 (5.3%)	-16.8 (3.2%)	-16.8 (2.8%)
ΔE_{elstat}[a]	-116.4 (39.8%)	-186.9 (30.4%)	-255.7 (37.3%)	-176.7 (55.8%)	-236.9 (45.1%)	-424.8 (70.1%)
ΔE_{orb}[a]	-159.1 (54.4%)	-411.3 (66.9%)	-412.8 (60.2%)	-123.4 (38.9%)	-271.5 (51.7%)	-164.8 (27.2%)

[a]The values in parentheses give the percentage contribution to the total attractive interactions $\Delta E_{elstat} + \Delta E_{orb} + \Delta E_{disp}$

Table S7. The Cartesian Coordinates and ZPE corrected electronic energies of the minimum energy geometries of $M(Bz)_3$ and $M(Bz)_2$ complexes at the M06-2X/def2-TZVPP level.

$Ca(Bz)_3$ (D_3, $^1A_1$)

E_0 = -1373.997563 au

C	-0.321125000	2.379615000	1.376212000
C	1.008941000	2.453387000	0.957154000
C	1.342327000	2.452140000	-0.388781000
C	0.321125000	2.379615000	-1.376212000
C	-1.008941000	2.453387000	-0.957154000
C	-1.342327000	2.452140000	0.388781000
H	-0.565848000	2.401998000	2.427059000
H	1.795164000	2.476400000	1.701104000
H	2.377559000	2.518444000	-0.687155000
H	0.565848000	2.401998000	-2.427059000
H	-1.795164000	2.476400000	-1.701104000
H	-2.377559000	2.518444000	0.687155000
C	1.620225000	-2.100461000	0.957154000
C	2.221369000	-0.911705000	1.376212000
C	2.794779000	-0.063580000	0.388781000
C	2.629165000	-0.352925000	-0.957154000
C	1.900244000	-1.467910000	-1.376212000
C	1.452452000	-2.388560000	-0.388781000
H	1.247043000	-2.792858000	1.701104000
H	2.363115000	-0.710961000	2.427059000
H	3.369816000	0.799805000	0.687155000
H	3.042208000	0.316458000	-1.701104000

H	1.797267000	-1.691037000	-2.427059000
H	0.992257000	-3.318249000	-0.687155000
C	-2.629165000	-0.352925000	0.957154000
C	-1.900244000	-1.467910000	1.376212000
C	-1.452452000	-2.388560000	0.388781000
C	-1.620225000	-2.100461000	-0.957154000
C	-2.221369000	-0.911705000	-1.376212000
C	-2.794779000	-0.063580000	-0.388781000
H	-3.042208000	0.316458000	1.701104000
H	-1.797267000	-1.691037000	2.427059000
H	-0.992257000	-3.318249000	0.687155000
H	-1.247043000	-2.792858000	-1.701104000
H	-2.363115000	-0.710961000	-2.427059000
H	-3.369816000	0.799805000	-0.687155000
Ca	0.000000000	0.000000000	0.000000000

$Sr(Bz)_3$ (D_3, $^1A_1$)

E_0 = -727.065827 au

C	-0.330718000	2.527046000	1.374701000
C	1.002865000	2.576050000	0.965694000
C	1.347261000	2.562728000	-0.380051000
C	0.330718000	2.527046000	-1.374701000
C	-1.002865000	2.576050000	-0.965694000
C	-1.347261000	2.562728000	0.380051000
H	-0.582820000	2.562081000	2.423641000
H	1.784739000	2.596758000	1.714664000
H	2.386411000	2.610309000	-0.670652000
H	0.582820000	2.562081000	-2.423641000
H	-1.784739000	2.596758000	-1.714664000

H	-2.386411000	2.610309000	0.670652000
C	1.729492000	-2.156532000	0.965694000
C	2.353845000	-0.977113000	1.374701000
C	2.893018000	-0.114602000	0.380051000
C	2.732357000	-0.419518000	-0.965694000
C	2.023127000	-1.549933000	-1.374701000
C	1.545757000	-2.448127000	-0.380051000
H	1.356489000	-2.844009000	1.714664000
H	2.510237000	-0.776304000	2.423641000
H	3.453800000	0.761538000	0.670652000
H	3.141228000	0.247250000	-1.714664000
H	1.927418000	-1.785777000	-2.423641000
H	1.067389000	-3.371847000	-0.670652000
C	-2.732357000	-0.419518000	0.965694000
C	-2.023127000	-1.549933000	1.374701000
C	-1.545757000	-2.448127000	0.380051000
C	-1.729492000	-2.156532000	-0.965694000
C	-2.353845000	-0.977113000	-1.374701000
C	-2.893018000	-0.114602000	-0.380051000
H	-3.141228000	0.247250000	1.714664000
H	-1.927418000	-1.785777000	2.423641000
H	-1.067389000	-3.371847000	0.670652000
H	-1.356489000	-2.844009000	-1.714664000
H	-2.510237000	-0.776304000	-2.423641000
H	-3.453800000	0.761538000	-0.670652000
Sr	0.000000000	0.000000000	0.000000000

$Ba(Bz)_3$ (D_3, $^1A_1$)

E_0 = -721.840749 au

C	-0.434658000	2.741328000	1.340680000
C	0.922727000	2.763690000	1.045234000
C	1.374029000	2.731667000	-0.275912000
C	0.434658000	2.741328000	-1.340680000
C	-0.922727000	2.763690000	-1.045234000
C	-1.374029000	2.731667000	0.275912000
H	-0.768403000	2.782945000	2.367350000
H	1.642304000	2.788637000	1.854273000
H	2.432541000	2.773661000	-0.487080000
H	0.768403000	2.782945000	-2.367350000
H	-1.642304000	2.788637000	-1.854273000
H	-2.432541000	2.773661000	0.487080000
C	1.932062000	-2.180950000	1.045234000
C	2.591388000	-0.994239000	1.340680000
C	3.052708000	-0.175889000	0.275912000
C	2.854789000	-0.582740000	-1.045234000
C	2.156730000	-1.747089000	-1.340680000
C	1.678678000	-2.555778000	-0.275912000
H	1.593879000	-2.816596000	1.854273000
H	2.794302000	-0.726016000	2.367350000
H	3.618332000	0.719812000	0.487080000
H	3.236183000	0.027958000	-1.854273000
H	2.025899000	-2.056929000	-2.367350000
H	1.185790000	-3.493473000	-0.487080000
C	-2.854789000	-0.582740000	1.045234000
C	-2.156730000	-1.747089000	1.340680000
C	-1.678678000	-2.555778000	0.275912000

C	-1.932062000	-2.180950000	-1.045234000
C	-2.591388000	-0.994239000	-1.340680000
C	-3.052708000	-0.175889000	-0.275912000
H	-3.236183000	0.027958000	1.854273000
H	-2.025899000	-2.056929000	2.367350000
H	-1.185790000	-3.493473000	0.487080000
H	-1.593879000	-2.816596000	-1.854273000
H	-2.794302000	-0.726016000	-2.367350000
H	-3.618332000	0.719812000	-0.487080000
Ba	0.000000000	0.000000000	0.000000000

Ca(Bz)$_3$ (C_1, ^{3}A)

E_0 = -1373.991507 au

C	-1.163859000	2.115258000	-1.397215000
C	-2.177766000	1.824873000	-0.463473000
C	-1.936015000	1.958932000	0.898330000
C	-0.695052000	2.387597000	1.365453000
C	0.285609000	2.785498000	0.428154000
C	0.048727000	2.646005000	-0.930479000
H	-1.348835000	2.029384000	-2.457841000
H	-3.146339000	1.490768000	-0.806232000
H	-2.711110000	1.702773000	1.608767000
H	-0.512571000	2.492954000	2.424714000
H	1.227848000	3.182584000	0.774511000
H	0.813458000	2.929632000	-1.640642000
C	-1.390219000	-2.118971000	-1.350816000
C	-2.448288000	-1.327108000	-0.923411000
C	-2.660443000	-1.098776000	0.434308000

C	-1.798100000	-1.660945000	1.387176000
C	-0.773552000	-2.513789000	0.959259000
C	-0.570462000	-2.748932000	-0.394265000
H	-1.226263000	-2.291287000	-2.404845000
H	-3.098392000	-0.864924000	-1.654318000
H	-3.489826000	-0.484450000	0.752652000
H	-1.959554000	-1.492855000	2.442354000
H	-0.128286000	-2.982075000	1.689822000
H	0.222698000	-3.405693000	-0.717678000
C	2.539312000	0.080417000	-1.332456000
C	2.257540000	-1.299143000	-1.063865000
C	2.289848000	-1.731582000	0.286601000
C	2.400143000	-0.832910000	1.328217000
C	2.558909000	0.565748000	1.059903000
C	2.716916000	0.968555000	-0.290808000
H	2.644938000	0.418445000	-2.354540000
H	2.196612000	-2.011633000	-1.872134000
H	2.196532000	-2.786589000	0.509778000
H	2.401181000	-1.187861000	2.350063000
H	2.720549000	1.262402000	1.867980000
H	2.954628000	2.000804000	-0.513141000
Ca	0.167163000	-0.000338000	-0.000148000

$Sr(Bz)_3$ (C_2, ^{3}A)

E_0 = -727.062218 au

C	1.653371000	1.820924000	-1.582663000
C	0.641185000	1.476207000	-2.496628000
C	-0.679903000	1.845340000	-2.251490000

C	-1.020566000	2.547338000	-1.099688000
C	0.000000000	2.936853000	-0.204086000
C	1.317566000	2.577581000	-0.451832000
H	2.683912000	1.563573000	-1.779662000
H	0.889196000	0.934976000	-3.398570000
H	-1.453948000	1.564981000	-2.954157000
H	-2.045766000	2.833956000	-0.915353000
H	-0.248240000	3.508100000	0.678412000
H	2.092594000	2.871493000	0.243372000
C	1.020566000	-2.547338000	-1.099688000
C	0.679903000	-1.845340000	-2.251490000
C	-0.641185000	-1.476207000	-2.496628000
C	-1.653371000	-1.820924000	-1.582663000
C	-1.317566000	-2.577581000	-0.451832000
C	0.000000000	-2.936853000	-0.204086000
H	2.045766000	-2.833956000	-0.915353000
H	1.453948000	-1.564981000	-2.954157000
H	-0.889196000	-0.934976000	-3.398570000
H	-2.683912000	-1.563573000	-1.779662000
H	-2.092594000	-2.871493000	0.243372000
H	0.248240000	-3.508100000	0.678412000
C	1.386124000	0.236453000	2.604432000
C	0.895297000	-1.106500000	2.538959000
C	-0.511048000	-1.303056000	2.622055000
C	-1.386124000	-0.236453000	2.604432000
C	-0.895297000	1.106500000	2.538959000
C	0.511048000	1.303056000	2.622055000
H	2.451658000	0.415402000	2.665410000

H	1.575094000	-1.942037000	2.605925000
H	-0.905376000	-2.309317000	2.689082000
H	-2.451658000	-0.415402000	2.665410000
H	-1.575094000	1.942037000	2.605925000
H	0.905376000	2.309317000	2.689082000
Sr	0.000000000	0.000000000	0.110063000

$Ba(Bz)_3$ (C_2, ^{3}A)

E_0 = -721.839730 au

C	0.000000000	2.564782000	-1.754577000
C	-0.556082000	1.606280000	-2.619864000
C	-1.807357000	1.055713000	-2.343325000
C	-2.507962000	1.424661000	-1.200251000
C	-1.954356000	2.389509000	-0.332488000
C	-0.717965000	2.956211000	-0.620670000
H	0.951820000	3.023441000	-1.981309000
H	-0.026948000	1.316363000	-3.517033000
H	-2.231462000	0.322554000	-3.017631000
H	-3.479085000	0.998411000	-0.992568000
H	-2.495328000	2.693090000	0.552815000
H	-0.302144000	3.700813000	0.045639000
C	2.507962000	-1.424661000	-1.200251000
C	1.807357000	-1.055713000	-2.343325000
C	0.556082000	-1.606280000	-2.619864000
C	0.000000000	-2.564782000	-1.754577000
C	0.717965000	-2.956211000	-0.620670000
C	1.954356000	-2.389509000	-0.332488000
H	3.479085000	-0.998411000	-0.992568000

H	2.231462000	-0.322554000	-3.017631000
H	0.026948000	-1.316363000	-3.517033000
H	-0.951820000	-3.023441000	-1.981309000
H	0.302144000	-3.700813000	0.045639000
H	2.495328000	-2.693090000	0.552815000
C	0.943944000	1.038273000	2.805024000
C	1.387888000	-0.314597000	2.739025000
C	0.402318000	-1.342845000	2.806797000
C	-0.943944000	-1.038273000	2.805024000
C	-1.387888000	0.314597000	2.739025000
C	-0.402318000	1.342845000	2.806797000
H	1.671921000	1.837252000	2.866629000
H	2.438410000	-0.551377000	2.813095000
H	0.712727000	-2.378485000	2.869473000
H	-1.671921000	-1.837252000	2.866629000
H	-2.438410000	0.551377000	2.813095000
H	-0.712727000	2.378485000	2.869473000
Ba	0.000000000	0.000000000	0.124388000

$Ca(Bz)_2$ (C_{2v}, $^1A_1$)

E_0 = -1141.842561 au

C	-1.224347000	1.879460000	-0.793900000
C	-1.218638000	2.361374000	0.496983000
C	0.000000000	2.517092000	1.222152000
C	1.218638000	2.361374000	0.496983000
C	1.224347000	1.879460000	-0.793900000
C	0.000000000	1.490823000	-1.435864000
H	-2.162280000	1.781628000	-1.323268000

H	-2.156003000	2.626788000	0.968690000
H	0.000000000	2.959214000	2.205869000
H	2.156003000	2.626788000	0.968690000
H	2.162280000	1.781628000	-1.323268000
H	0.000000000	1.233256000	-2.481591000
C	-1.224347000	-1.879460000	-0.793900000
C	-1.218638000	-2.361374000	0.496983000
C	0.000000000	-2.517092000	1.222152000
C	1.218638000	-2.361374000	0.496983000
C	1.224347000	-1.879460000	-0.793900000
C	0.000000000	-1.490823000	-1.435864000
H	-2.162280000	-1.781628000	-1.323268000
H	-2.156003000	-2.626788000	0.968690000
H	0.000000000	-2.959214000	2.205869000
H	2.156003000	-2.626788000	0.968690000
H	2.162280000	-1.781628000	-1.323268000
H	0.000000000	-1.233256000	-2.481591000
Ca	0.000000000	0.000000000	0.583016000

$Sr(Bz)_2$ (C_{2v}, $^1A_1$)

E_0 = -494.899673 au

C	-1.224998000	1.950854000	-0.929598000
C	-1.218549000	2.540602000	0.316065000
C	0.000000000	2.764532000	1.017206000
C	1.218549000	2.540602000	0.316065000
C	1.224998000	1.950854000	-0.929598000
C	0.000000000	1.520242000	-1.537561000
H	-2.161982000	1.812936000	-1.451970000

H	-2.155253000	2.848545000	0.763148000
H	0.000000000	3.290272000	1.959493000
H	2.155253000	2.848545000	0.763148000
H	2.161982000	1.812936000	-1.451970000
H	0.000000000	1.184452000	-2.560864000
C	-1.224998000	-1.950854000	-0.929598000
C	-1.218549000	-2.540602000	0.316065000
C	0.000000000	-2.764532000	1.017206000
C	1.218549000	-2.540602000	0.316065000
C	1.224998000	-1.950854000	-0.929598000
C	0.000000000	-1.520242000	-1.537561000
H	-2.161982000	-1.812936000	-1.451970000
H	-2.155253000	-2.848545000	0.763148000
H	0.000000000	-3.290272000	1.959493000
H	2.155253000	-2.848545000	0.763148000
H	2.161982000	-1.812936000	-1.451970000
H	0.000000000	-1.184452000	-2.560864000
Sr	0.000000000	0.000000000	0.655976000

$Ba(Bz)_2$ (C_{2v}, $^1A_1$)

E_0 = -489.677220 au

C	-1.225139000	2.038604000	-1.088797000
C	-1.218283000	2.723263000	0.107785000
C	0.000000000	3.008134000	0.777405000
C	1.218283000	2.723263000	0.107785000
C	1.225139000	2.038604000	-1.088797000
C	0.000000000	1.574960000	-1.660554000
H	-2.161700000	1.854882000	-1.597851000

H	-2.154585000	3.061615000	0.533369000
H	0.000000000	3.598978000	1.681149000
H	2.154585000	3.061615000	0.533369000
H	2.161700000	1.854882000	-1.597851000
H	0.000000000	1.147739000	-2.649625000
C	-1.225139000	-2.038604000	-1.088797000
C	-1.218283000	-2.723263000	0.107785000
C	0.000000000	-3.008134000	0.777405000
C	1.218283000	-2.723263000	0.107785000
C	1.225139000	-2.038604000	-1.088797000
C	0.000000000	-1.574960000	-1.660554000
H	-2.161700000	-1.854882000	-1.597851000
H	-2.154585000	-3.061615000	0.533369000
H	0.000000000	-3.598978000	1.681149000
H	2.154585000	-3.061615000	0.533369000
H	2.161700000	-1.854882000	-1.597851000
H	0.000000000	-1.147739000	-2.649625000
Ba	0.000000000	0.000000000	0.720303000
